# Prognostic implications of adverse events associated with CAR-T cell therapy: a population-based global observational study

**DOI:** 10.1016/j.eclinm.2025.103623

**Published:** 2025-11-03

**Authors:** Zhen Sun, Jianglong Guo, Mengsi Liu, Hongye Wang, Zhe Li, Weiming Shen, Siying Wang, Huijue Zhu, Xiaoye Liu, Jinhao Li, Yuan Ouyang, Yueze Zhu, Zhen Ye, Shunpeng Xing, Gang Chen, Haojie Jin

**Affiliations:** a State Key Laboratory of Systems Medicine for Cancer, Shanghai Cancer Institute, Renji Hospital, Shanghai Jiao Tong University School of Medicine, Shanghai, China; b Guangdong Cardiovascular Institute, Guangzhou, China; c Department of Biliary and Pancreatic Surgery, Renji Hospital, Shanghai Jiao Tong University School of Medicine, Shanghai, China; d Hengyang Medical School, University of South China, Hengyang, China; e The First Affiliated Hospital of University of South China, Hengyang, China; f Department of Critical Care Medicine, Renji Hospital, Shanghai Jiao Tong University School of Medicine, Shanghai, China; g Zhejiang Key Laboratory of Intelligent Cancer Biomarker Discovery & Translation, Department of Hepatopancreatobiliary Surgery, The First Affiliated Hospital of Wenzhou Medical University, Wenzhou, China

**Keywords:** Chimeric antigen receptor T-cell therapy, Adverse event, Drug safety, Fatality, Prognosis, Big data

## Abstract

**Background:**

Chimeric antigen receptor (CAR)-T cell therapy offers a promising and transformative treatment option for patients with hematologic malignancies, with expanding potential in solid tumors and non-malignant diseases. However, it also exposes recipients to a wide spectrum of treatment-related toxicities, complicating its clinical implementation. The limited sample sizes of clinical studies hinder a comprehensive understanding of how different adverse events (AEs) may impact CAR-T treatment outcomes.

**Methods:**

Based on two global real-world drug safety surveillance systems, this observational pharmacovigilance study identified safety signals and death-related AEs in CAR-T cell therapy. All consecutive CAR-T cell-treated cases with AEs reported to the World Health Organization’ VigiBase (as of March 2025) and U.S. Food and Drug Administration Adverse Event Reporting System (as of June 2024) were included. We evaluated disproportionate risk and death of all reported AEs by the modified reporting odds ratio method. AE fatality rates, reporting odds ratio of fatality rates among different AEs, and CAR-T cell therapy safety signals were reported.

**Findings:**

This analysis included 12,511 CAR-T cell-treated cases in FAERS from 37 countries/regions, 2861 had a fatal outcome. Disproportionality analysis identified 266 AEs as safety signals associated with CAR-T therapy. Of these, 59 high-fatality AEs were revealed (average fatality rate 49.35% [189/383]), such as pulmonary hemorrhage, lactic acidosis, and hemophagocytic lymphohistiocytosis. Cardiac events and respiratory/infection-related complications showed notably high fatality and high reporting frequencies, respectively. We identified 31 low-fatality AEs indicating a comparatively better outcome (average fatality: 11.47% [710/6188]), such as cytokine release syndrome, bradyphrenia, and hypocalcemia, which were predominantly neurologic or immune-related. These prognostically significant AEs were also borne out as death signals for CAR-T cell administration in 5555 CAR-T cell-treated cases from VigiBase.

**Interpretation:**

This work establishes a systematic mapping of prognostically significant AEs in CAR-T cell therapy, providing a data-driven resource to inform future research and toxicity management strategies. Given the inherent limitations in pharmacovigilance data, including potential reporting bias and temporal ambiguities, the identified signals require further research to validate causality.

**Funding:**

This study was funded by the 10.13039/501100012166National Key Research and Development Program of China (2022YFC2804300), the 10.13039/501100001809National Natural Science Foundation of China (82222047, W2411079), the 10.13039/501100003399Science and Technology Commission of Shanghai Municipality (22XD1423100), and the 10.13039/100017950Shanghai Municipal Health Commission (2022XD057, 2024ZZ1008).

**Research in context**

**Evidence before this study**

We conducted a systematic literature search in PubMed and Web of Science up to July 20, 2025, using the following combination of terms: (“adverse events” or “adverse drug reactions” or “treatment toxicity”), (“chimeric antigen receptor T-cell therapy” or “CAR-T cell” or “adoptive cell therapy”), (“death” or “fatal outcome” or “prognosis”), and “real world pharmacovigilance”. This search yielded five publications. However, these publications focused on characterizing the general toxicity profile of CAR-T therapy, without leveraging large-scale real-world pharmacovigilance data to assess the associations between specific AEs and patient outcomes. Overall, the prognostic implications of different CAR-T-related AEs on clinical outcomes remains insufficiently explored and lacks systematic evaluation.

**Added value of this study**

Compared to previous pharmacovigilance studies, the unique contribution of this work is its two-stage analytic framework, which builds on conventional treatment-related safety signals and further identifies signals related to patient outcomes. Based on two large-scale pharmacovigilance surveillance systems, this study overcomes the challenges posed by the limited data available in clinical studies, enabling systematic evaluation of the prognostic significance of various AEs in CAR-T cell therapy. To our knowledge, this represents the most comprehensive and highest granularity analysis to date into the downstream survival implications of AEs associated with CAR-T therapy, revealing a group of events that lead to excess fatal or better outcomes, defined as the “prognostically significant adverse event spectrum”.

**Implications of all the available evidence**

This study identified AEs that are not only associated with CAR-T therapy but also correlated with patient outcomes, providing a systematic and data-driven resource for better understanding the impact of various AEs on prognosis. Many high-fatality events reaffirm established risks, and we further quantified these associations within the context of CAR-T-specific safety signals. Additionally, the findings reveal potential opportunities for targeted interventions. For instance, the study uncovers new hypotheses regarding less-recognized AEs, such as hypocalcemia, which is associated with comparatively better outcome, and hypercalcemia, which correlates with higher mortality. We also observed that immune effector cell-associated neurotoxicity syndrome shows opposite prognostic implications in anti-CD19 and anti-BCMA CAR-T therapies, suggesting that antigen targets and baseline disease also play an important role in the relationship between toxicity and outcome. These results encourage the future integration of AE profiles with established prognostic biomarkers to enhance patient management, trial design, and CAR-T cell optimization research. Furthermore, given the overreporting of certain highly fatal AEs such as serious cardiac and infection-related events in CAR-T therapy, formal contraindications may be needed for patients at high risk for these complications.

## Introduction

Chimeric antigen receptor (CAR)-T cell therapy is a transformative advance in immunotherapy, utilizing genetic engineering to reprogram autologous or allogeneic T cells to specifically target cancer cells, independent of the major histocompatibility complex.^1^ CAR-T cell therapy shows unprecedented curative potential, and is rapidly changing the treatment paradigm for hematologic malignancies. Despite this promise, CAR-T cell treatments are often accompanied by complex and diverse side effects.^2^^,^^3^

Adverse events (AEs) complicate treatment and may interact with the effects of CAR-T cells, affecting patient survival. Previous studies have suggested a potential link between CAR-T cell toxicity and response. For instance, cytokine release syndrome (CRS), a CAR-T cell therapy-specific AE, is viewed as a marker of CAR-T cell activation and expansion, potentially correlating with favorable clinical outcomes.^4^, ^5^, ^6^ Conversely, cardiac complications may increase the risk of non-relapse mortality.^7^ Although clinical studies have provided some evidence linking a few specific AEs to clinical outcomes, the small sample sizes limit the possibility of systematically exploring these associations across the full spectrum of AEs.^8^, ^9^, ^10^ Current knowledge gaps prevent clinicians from offering personalized advice and case management based on AEs experienced by patients. Real-world AE data from large pharmacovigilance systems can provide insights that cannot be captured in clinical studies, due to their broad data coverage and more diverse population.^11^^,^^12^ Although there have been some pharmacovigilance analyses on CAR-T therapy to understand its toxicity and characteristics, very few studies have focused on how different treatment-related AEs impact patient outcomes.^13^, ^14^, ^15^, ^16^

In this study, we used data from two global wide-scale pharmacovigilance systems, and performed modified disproportionality analysis to comprehensively investigate the prognostic implications of AEs following CAR-T cell therapy. This is the first systematic mapping of AEs with prognostic significance in CAR-T cell therapy using pharmacovigilance data. Prognostically significant AEs were categorized into high-fatality and low-fatality AEs. These findings provide a preliminary framework for distinguishing AEs associated with distinct clinical outcomes to improve the success of CAR-T cell therapy.

## Methods

### Data sources

This is an observational, real-world pharmacovigilance study based on large-scale drug safety surveillance databases. VigiBase, the World Health Organization (WHO) global database of reported adverse events of medicinal products, comprises more than 40 million Individual Case Safety Reports (ICSRs) submitted by over 180 member countries or territories of the WHO Programme for International Drug Monitoring. The U.S. Food and Drug Administration (FDA) Adverse Event Reporting System (FAERS), a safety surveillance program, includes worldwide post-marketing AE data for therapeutic products submitted to the FDA. Both VigiBase and FAERS include mandatory reports from manufacturers as well as voluntary submissions from healthcare professionals and consumers. We extracted all available safety reports from FAERS up to June 2024 and from VigiBase up to March 2025.

Six commercially available CAR-T cell products approved by the FDA were included: idecabtagene vicleucel, lisocabtagene maraleucel, ciltacabtagene autoleucel, tisagenlecleucel, brexucabtagene autoleucel, and axicabtagene ciloleucel. For FAERS, we identified relevant safety reports using the generic names, brand names, and other synonyms of the above CAR-T cell therapies. For VigiBase, as drug entries are coded according to WHODrug standards, we identified reports using the corresponding *Medicinalprod_Id* codes for each CAR-T cell product. To ensure specificity of signal detection, we only analyzed cases in which a CAR-T cell product was identified as the primary suspected drug for AEs. Since VigiBase does not distinguish between primary and secondary suspect roles, all reports in which a CAR-T cell product was labeled as “suspect” were included.

### Ethics

The WHO's VigiBase is accessible upon request through the Uppsala Monitoring Centre, and the U.S. FAERS is publicly available. Patient records available in both VigiBase and FAERS are entirely de-identified and anonymized; therefore, ethical approval and written informed consent are exempted for this study.

### Adverse events, patient outcomes, and determination of sex

Adverse drug reactions were standardized and coded using the Medical Dictionary for Regulatory Activities Terminology (MedDRA, version 26.1, www.meddra.org) at the preferred term (PT) level, and each PT could be classified into a primary System Organ Class (SOC). PTs across eighteen organ systems were evaluated to determine if they were related to CAR-T cell therapy and patient outcome (Supplementary Table S1). Analyzing adverse event signals at the PT level is standard practice in pharmacovigilance, as it provides the necessary granularity and clinical relevance to distinguish and classify events. However, this approach may introduce some redundancy in terminology. To improve clarity, we also reported the hierarchical relationship between PTs and the higher MedDRA levels, including High Level Term (HLT) and High Level Group Term (HLGT).

In the FAERS and VigiBase databases, all submitted reports include outcome information. If the outcome is reported as severe, it is further categorized into “death”, “life threatening”, “caused/prolonged hospitalization”, “disabling/Incapacitating”, “congenital anomaly/birth defect”, “required intervention to prevent permanent impairment/damage”, or “other”. In our study, fatality is defined as the reported outcome of “death”. This definition reflects the all-caused death as recorded by the reporter, without determining whether death was directly caused by the AEs, underlying disease, or other factors.

The sex of patients in both the FAERS and VigiBase databases is recorded based on self-report or the healthcare provider's report, and is classified as either male or female. While the methods for determining sex are not always explicitly stated, it is generally assumed that sex is recorded according to the patient's biological characteristics as reported.

### Data processing workflow

In FAERS, for cases with multiple submissions, only the most recent follow-up version was retained. Subsequently, for distinct case numbers, we screened for consistency across the following key variables to reduce the inclusion of potentially duplicate reports: age, sex, country of occurrence, event date, drug, AE, indication, and outcome. In VigiBase, duplicate cases were removed using the automatic deduplication algorithm, vigiMatch. Notably, VigiBase includes ICSRs from the United States that are also submitted to FAERS; these cases were excluded from the VigiBase dataset and retained only in the FAERS dataset. Given the relatively large amount of data available in FAERS, our primary analysis was based on FAERS data, while the VigiBase database was used to validate the findings.

### Statistics

In the field of pharmacovigilance, disproportionality analysis is the primary and preferred tool for detecting potential associations between specific AEs and drugs of interest from large-scale surveillance databases.^17^^,^^18^ Specifically, this method determines whether a drug-AE combination occurs more frequently than expected by comparing the proportion of an AE following CAR-T cell treatment (cases) to the proportion of the same AE occurring with other drugs in the entire database (non-cases), also known as case-non-case analysis. In this study, we used the reporting odds ratio (ROR) method, a frequentist approach that is the pharmacovigilance equivalent of the odds ratio and can be easily interpreted. The ROR method has been shown to provide the most comprehensive safety signal and estimate relative risk.^19^^,^^20^ For each AE, we constructed a 2 × 2 contingency table, based on which we calculated the ROR and corresponding 95% confidence intervals (CIs) using the following formula:

$$\text{ROR}=\frac{\left(a/c\right)}{\left(b/d\right)}$$

$$\text{ROR}025=e^{\ln\left(\text{ROR}\right)\;-\;1.96\sqrt{\;\left(\frac{1}{a}+\frac{1}{b}+\frac{1}{c}+\frac{1}{d}\right)\;}}$$

$$\text{ROR}975=e^{\ln\left(\text{ROR}\right)\;+\;1.96\sqrt{\;\left(\frac{1}{a}+\frac{1}{b}+\frac{1}{c}+\frac{1}{d}\right)\;}}$$

ROR025 refers to the 2.5th percentile of the ROR, representing the lower bound of 95% CIs, while ROR975 refers to the 97.5th percentile, representing the upper bound. To assess the impact of AEs on patient outcomes, the analytic process consisted of two phases. In the first phase, we screened all AEs reported during CAR-T cell therapy based on a drug-AE contingency table to identify CAR-T therapy-specific safety signals (Supplementary Table S2). To ensure a balanced comparison, non-CAR-T comparator group included patients with lymphoma, lymphoblastic leukemia, and plasma cell myeloma, based on indication information in the safety reports. These cancer types are similar to the approved indications for CAR-T therapies. A positive signal was defined by an ROR025 value greater than 1 and at least three reported cases of the corresponding AE, which is a conventional threshold in pharmacovigilance studies.^21^, ^22^, ^23^ In the second stage, the analysis was restricted to CAR-T cell-treated cases, which were categorized based on reported outcomes into fatal and non-fatal endpoints. We compared the fatality rate of each AE with that of other AEs using the ROR method based on patient outcome-AE contingency table (Supplementary Table S3). High-fatality AEs were defined as those with ROR025 > 1 and at least 3 fatal cases. For low-fatality AEs, ROR975 < 1 and at least 3 non-fatal cases were required. Next, we filtered high- and low-fatality AEs by using the CAR-T cell therapy safety signals identified in the first stage. This process aimed to identify CAR-T cell therapy related high-fatality/low-fatality AEs, which were interpreted as having a potential association with CAR-T cell therapy and affecting patient outcomes. Furthermore, we performed stratified analyses by age, sex, drug, and indication to explore the prognostic significance of AEs across different patient subgroups. For combinations with zero values in the contingency table, we applied the Haldane-Anscombe correction, adding 0.5 to the a, b, c, and d cells to avoid calculation errors. All signal detection analyses did not apply adjustments for multiple comparisons, and events meeting the predefined thresholds were considered positive signals. Furthermore, we examined whether the identified prognostic CAR-T therapy safety signals were previously unrecognized by screening the FDA-provided Package Insert and Medication Guide and conducting PubMed searches using relevant terms.

Patients were categorized into four groups based on the type of AEs they experienced: high-fatality AE, low-fatality AE, concurrent high-fatality and low-fatality AEs, and other AE (OT-AE, those not classified as high-fatality, low-fatality, or CAR-T-specific events) groups. The fatality rates among these four groups were compared using the Chi-square test, as the total sample size for all comparisons exceeded 40 and the expected counts in every cell were above 5. To account for the influence of reporting time, the Cox proportional hazards regression model was applied to compare the outcomes across these groups, with the OT-AE group serving as the reference. The time scale was defined as the duration from the initiation of CAR-T cell therapy to reporting of AEs to FAERS or the manufacturer, whichever occurred first. Available clinical and demographic factors, including age, sex, cancer type, drug name, drug target, country of occurrence, and reporter occupation, were adjusted to control for potential confounders. Age, being the only quantitative variable, was treated as continuous, with missing values imputed using the median. An indicator variable was used for missing categorical data. Cox regression analyses were not performed on the VigiBase dataset due to the lack of a specific treatment start date to calculate the time from infusion to reporting. For VigiBase, the minimum and maximum possible time to onset of AEs was provided based on the precision of the reported treatment start and event occurrence dates. The average of the minimum and maximum onset times was used to estimate the specific time to onset ((TimeToOnsetMin + TimeToOnsetMax)/2). The log-rank test was used to compare time to onset among high-fatality, low-fatality, and OT-AEs. For tests with multiple pairwise comparisons, including the Chi-square test for fatality and the log-rank test for time to onset, the Bonferroni adjustment was applied to adjust p-values. All tests were two-tailed and p-value <0.05 was considered statistically significant. Statistical analyses and visualizations were performed using R software (version 4.3.1) with the following R packages: tidyr (version 1.3.1), dplyr (version 1.1.4), ggplot2 (version 3.5.1), descriptio (version 1.4.1), stringr (version 1.5.1), gtsummary (version 2.0.0), survival (version 3.5–7), and ggpubr (version 0.6.0).

### Role of the funding source

The funders of the study had no role in study design, data collection, data analysis, data interpretation, or writing of the report.

## Results

**Fig. 1:**
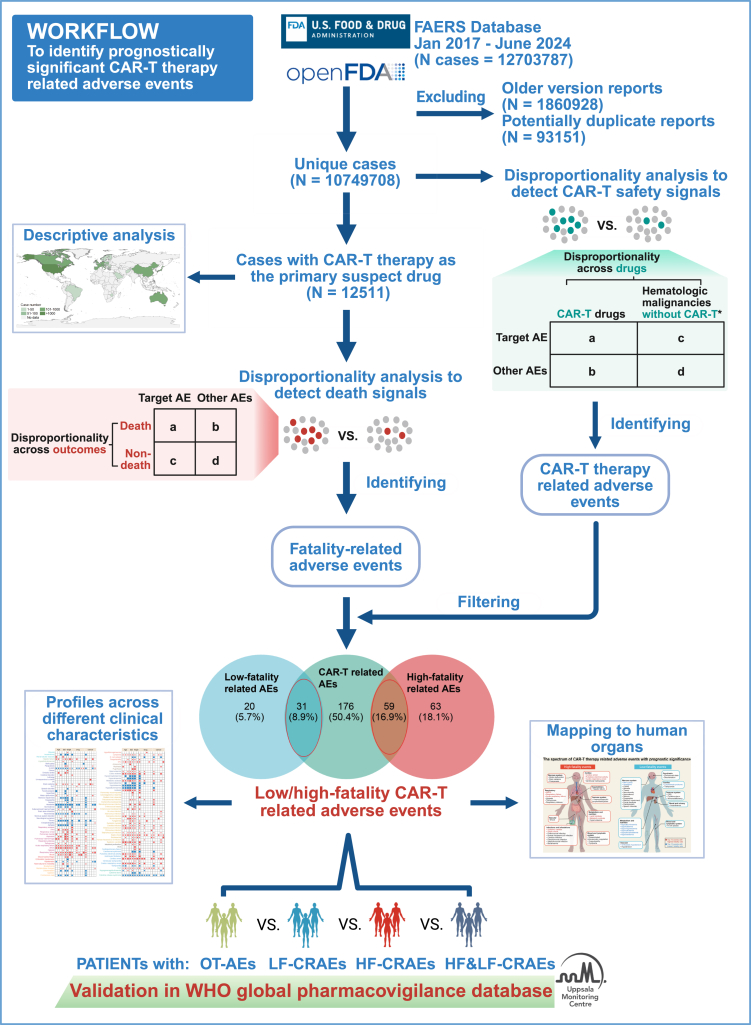
**Overview of the study design**. After screening for unique safety reports, a two-step analytic process was performed. In Phase 1, we systematically identified CAR-T therapy related adverse events. In Phase 2, we identified adverse events associated with the death outcome. The results of the two phases were intersected to identify high-fatality and low-fatality CAR-T therapy related adverse events. Subsequently, CAR-T cell treated cases were stratified into four groups based on their AE profiles: HF-CRAE, LF-CRAE, HF&LF-CRAE, and OT-AE groups. Comparative outcome analyses were performed across these groups and validated in the VigiBase database. ∗ The hematologic malignancies included lymphoma, lymphoblastic leukemia, and plasma cell myeloma. CAR, chimeric antigen receptor; FAERS, Food and Drug Administration Adverse Event Reporting System; WHO, World Health Organization; ROR, reporting odds ratio; LF-CRAEs, low-fatality CAR-T therapy related adverse events; HF-CRAEs, high-fatality CAR-T therapy related adverse events; HF&LF-CRAEs, concurrent high-fatality and low-fatality CAR-T therapy related adverse events; OT-AEs, other adverse events.

From January 2017 (the year the first CAR-T cell therapy product was approved by the FDA) through June 2024, FAERS received 12,703,787 safety reports, which included 41,444,087 AEs. After eliminating possible duplicates, 10,749,708 unique cases remained. Of these, CAR-T therapy was reported as the primary suspected drug in 12,511 cases (Fig. 1). As of March 2025, VigiBase contained 40,973,799 ICSRs. After excluding non-CAR-T reports, duplicates, and those originating from FAERS, 5555 CAR-T cell-treated cases from VigiBase were included in our analysis.

**Fig. 2:**
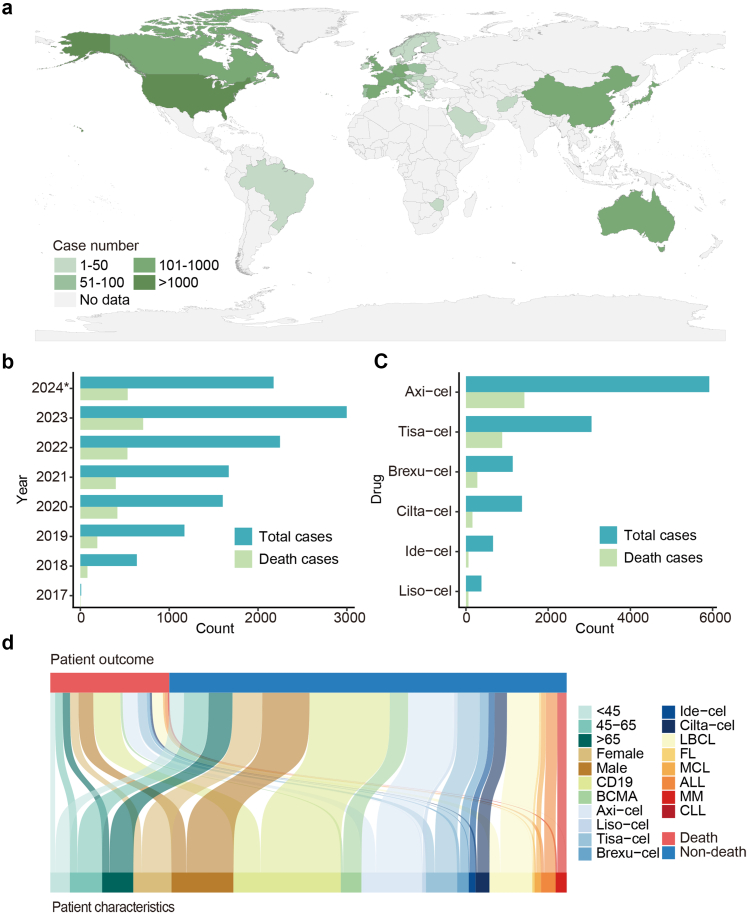
**Characteristics of safety reports with CAR-T cell therapy as the primary suspected drug in the FAERS database from 2017 to 2024**. (a) The numbers of cases reported according to country/region. (b) The numbers of reported cases and death outcomes per year. (c) The numbers of reported cases and death outcomes according to drug used. (d) Distribution of clinical characteristics according to outcomes. ∗ As of June 2024. CAR, chimeric antigen receptor; Axi-cel, Axicabtagene ciloleucel; Liso-cel, Lisocabtagene maraleucel; Tisa-cel, Tisagenlecleucel; Brexu-cel, Brexucabtagene autoleucel; Ide-cel, Idecabtagene vicleucel; Cilta-cel, Ciltacabtagene autoleucel; LBCL, large B-cell lymphoma; FL, follicular lymphoma; MCL, mantle cell lymphoma; ALL, acute lymphocytic leukemia; MM, multiple myeloma; CLL, chronic lymphocytic leukemia.

We developed a framework for using real-world pharmacovigilance data to analyze whether AEs of CAR-T cell therapies were associated with patient outcomes, and applied it to study outcome patterns for toxicities occurring in 12,511 patients in the FAERS database (Fig. 1). CAR-T cell safety reports came from 37 countries/regions, with the largest number from the United States (62.16%, 7777/12,511) (Fig. 2a). Since 2017, the number of CAR-T cell therapy-related safety reports has increased annually, corresponding to the growing clinical use of this therapy (Fig. 2b). The proportion of fatal outcomes is relatively stable. The largest proportion of cases were treated with axicabtagene ciloleucel (47.31%, 5919/12,511), followed by tisagenlecleucel (24.42%, 3055/12,511), ciltacabtagene autoleucel (10.89%, 1363/12,511), brexucabtagene autoleucel (9.10%, 1138/12,511), and idecabtagene vicleucel (5.27%, 659/12,511) (Fig. 2c). The number of safety reports for different CAR-T cell products was related to their approval timelines and market share. Most cases were reported by healthcare professionals (80.00%, 10,009/12,511). Among patients with available diagnostic information, 66.64% (5035/7556) had non-Hodgkin's lymphoma (including large B-cell, mantle cell, and follicular lymphomas). Other indications included acute lymphocytic leukemia (18.98%, 1434/7556), multiple myeloma (14.04%, 1061/7556), and chronic lymphocytic leukemia (0.34%, 26/7556). Median age was 61 years (interquartile range: 47–69), and the male-to-female ratio was 1.63 (6064/3729) (Supplementary Table S4). Fig. 2d shows the distribution of outcomes among patients with different characteristics.

The 12,511 CAR-T safety reports in FAERS included 1721 specific AEs. The non-CAR-T comparator group consisted of 499,737 hematologic cancer patients with 1,277,058 reported AEs, and the most frequently used drugs in this group are listed in Supplementary Table S5. Using this hematologic cancer cohort as a background, the ROR method identified 266 AEs as safety signals for CAR-T cell therapy, based on their disproportionately increased reporting frequencies following CAR-T cell administration (Supplementary Table S6). We then analyzed the association between these 266 CAR-T cell-related AEs and patient outcomes.

**Fig. 3:**
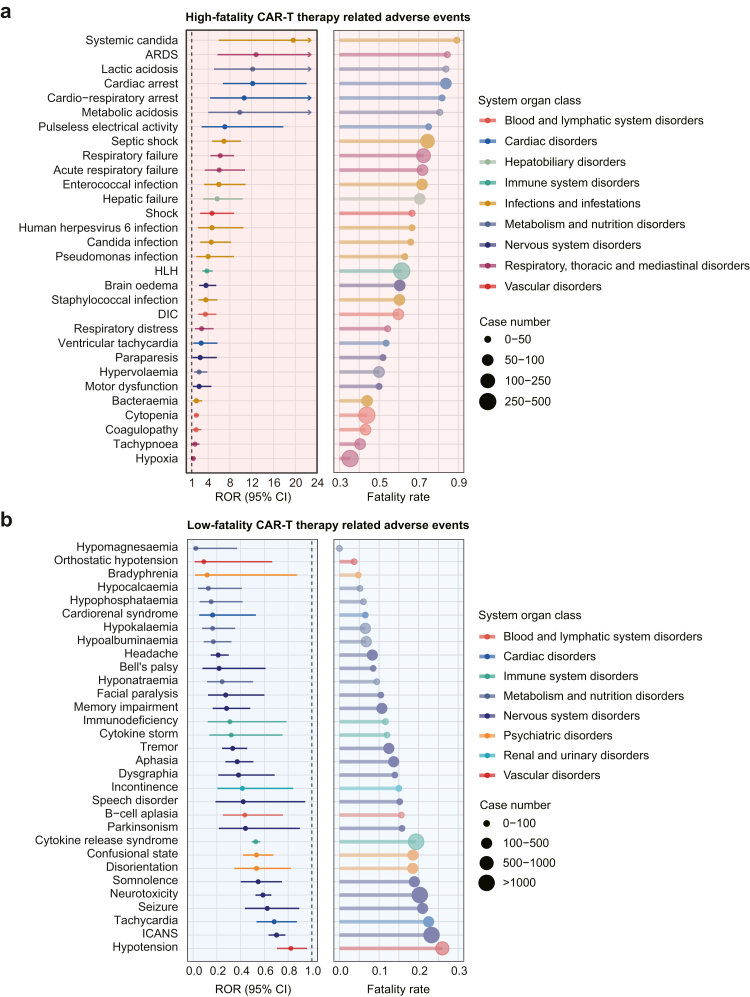
**The reporting odds ratios and case numbers for high-fatality and low-fatality CAR-T therapy related adverse events at the preferred term level**. (a) The top 30 high-fatality CAR-T therapy related adverse events according to occurrences. (b) The low-fatality CAR-T therapy related adverse events. In the left panel, the dots represent the reporting odds ratios, and error bars indicate the 95% confidence intervals. The right panel shows a histogram representing the fatality rates, with dots indicating the number of occurrences for each event. CAR, chimeric antigen receptor; ROR, reporting odds ratio; CI, confidence interval; ARDS, Acute respiratory distress syndrome; HLH, hemophagocytic lymphohistiocytosis; DIC, disseminated intravascular coagulation; ICANS, immune effector cell-associated neurotoxicity syndrome.

**Fig. 4:**
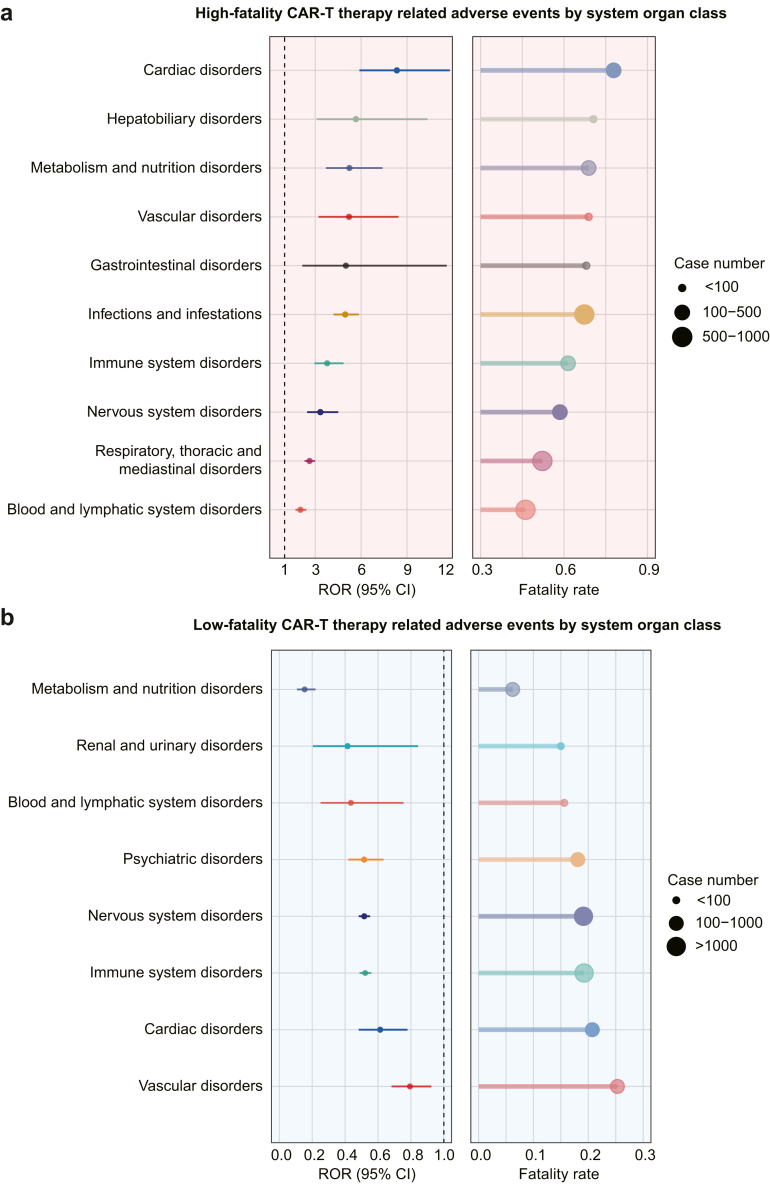
**The reporting odds ratios and case numbers for high-fatality and low-fatality CAR-T therapy related adverse events at the organ-system level**. (a) The high-fatality CAR-T therapy related adverse events at the organ-system level, showing the top 10 according to occurrences. (b) The low-fatality CAR-T therapy related adverse events at the organ-system level. In the left panel, the dots represent the reporting odds ratios, and error bars indicate the 95% confidence intervals. The right panel shows a histogram representing the fatality rates, with dots indicating the number of occurrences for each type of organ system. CAR, chimeric antigen receptor; ROR, reporting odds ratio; CI, confidence interval.

Among CAR-T cell-related events, 59 AEs were associated with excess fatality, occurring in 14.68% (1837/12,511) of CAR-T cell treated cases (Supplementary Fig. S1a and b). Among the top 30 most reported AEs, the five AEs with the highest fatality rates were systemic candida (ROR: 19.64; 95% CI: 5.93–65.06), acute respiratory distress syndrome (ROR: 12.81; 95% CI: 5.72–28.68), lactic acidosis (ROR: 12.18; 95% CI: 5.08–29.20), cardiac arrest (ROR: 12.18; 95% CI: 6.72–22.07), and cardio-respiratory arrest (ROR: 10.61; 95% CI: 4.38–25.70). The five most frequently reported AEs were hypoxia (23.68%, 435/1837), cytopenia (18.51%, 340/1837), hemophagocytic lymphohistiocytosis (HLH, 14.26%, 262/1837), respiratory failure (9.09%, 167/1837), and septic shock (7.89%, 145/1837) (Fig. 3a; Supplementary Table S7). AEs with high fatality were predominantly related to respiratory disorders (50.35%, 925/1837), infectious diseases (34.30%, 630/1837), and blood system disorders (27.49%, 505/1837) (Fig. 4a; Supplementary Table S8). High-fatality AEs were also observed in the cardiac, vascular, renal, neurological, metabolic, gastrointestinal, immune, eye, and hepatobiliary systems. Among the top 10 systems by number of reports, cardiac events had the highest fatality rate (ROR: 8.34; 95% CI: 5.89–11.80). Supplementary Figure S2 shows the MedDRA hierarchical relationship, with high-fatality CAR-T cell therapy-related AEs categorized under 38 HLT terms and 25 HLGT terms. Notably, some preferred terms may reflect the same clinical event. For example, respiratory distress, pulmonary alveolar hemorrhage, tachypnea, hypoxia, pulmonary hemorrhage, and acute respiratory distress syndrome could all be associated with respiratory failure; septic shock, shock, disseminated intravascular coagulation, and coagulopathy may result from severe infections or other causes that lead to circulatory system disturbances. A range of infectious diseases, including enterococcal infection, candida infection, fungaemia, mucormycosis, and adenovirus infection point to a burden of systemic and atypical infections during CAR-T therapy. Among the identified high-fatality AEs, hemorrhagic shock and lactic acidosis were not previously documented in the literature or product labels as direct safety signals of CAR-T cell therapy. However, these events are likely manifestations or complications of recognized CAR-T cell related AEs, such as HLH and infections.

Among 266 safety signals for CAR-T cell therapy, disproportionality analysis identified 31 low-fatality AEs, which were reported in 61.08% (7642/12,511) of cases (Supplementary Figure S1). Patients experiencing these AEs had comparatively superior outcomes, with RORs for fatality ranging from 0.02–0.82. The top five AEs with the lowest fatality rates were hypomagnesemia (ROR: 0.02; 95% CI: 0.00–0.37), orthostatic hypotension (ROR: 0.09; 95% CI: 0.01–0.67), bradyphrenia (ROR: 0.12; 95% CI: 0.02–0.88), hypocalcemia (ROR: 0.13; 95% CI: 0.04–0.41), and hypophosphatemia (ROR: 0.15; 95% CI: 0.06–0.42). The five low-fatality events with the highest number of cases were CRS (75.94%, 5803/7642), immune effector cell-associated neurotoxicity syndrome (ICANS, 29.09%, 2223/7642), neurotoxicity (24.95%, 1907/7642), hypotension (11.38%, 870/7642), and confusional state (5.99%, 458/7642) (Fig. 3b; Supplementary Table S9). Low-fatality AEs were primarily distributed in the nervous system (80.18%, 6127/7642) and the immune system (77.15%, 5896/7642), largely attributed to CRS and neurotoxicity (Fig. 4b; Supplementary Table S10). Low-fatality AEs also occurred across the psychiatric, hematologic, vascular, cardiac, renal, and metabolic systems. Metabolic and nutrition-related AEs had the lowest fatality rate (ROR: 0.15; 95% CI: 0.11–0.22). The higher MedDRA hierarchical relationships for low-fatality CAR-T cell-related adverse events are shown in Supplementary Figure S3. Many of these events reflect specific symptoms of neurotoxicity or ICANS, such as tremor, aphasia, dysgraphia, confusional state, and disorientation. In addition, hypotension and cytokine storm represent a possible manifestation of CRS and another term expression, respectively. Cardiorenal syndrome is closely associated with hypotension, tachycardia, and metabolism-related AEs. Among the low-fatality AEs, incontinence was identified as a previously unrecognized potential safety signal for CAR-T cell therapy, possibly a consequence of neurotoxicity.

**Fig. 5:**
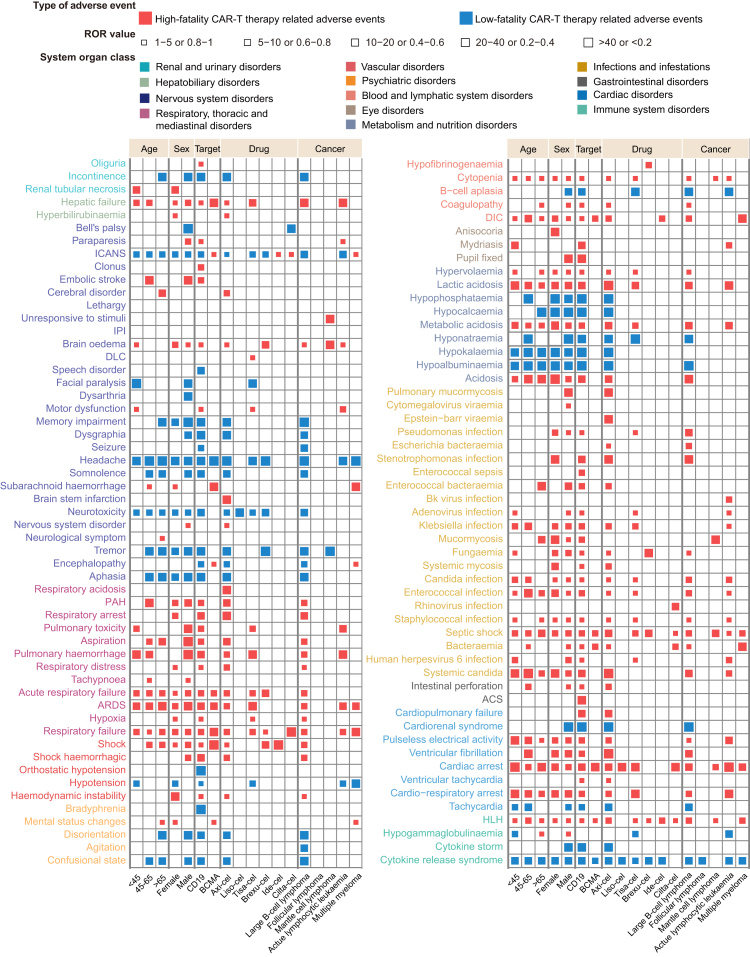
**Distribution of CAR-T therapy related death signals across different subpopulations**. Red squares represent specific CAR-T therapy related adverse events that are high-fatality signals for a certain subgroup, meaning that the events are related to significantly increased fatality. Blue squares represent specific CAR-T therapy related adverse events that are low-fatality signals for a certain subgroup. Blank boxes indicate that no fatality signal was detected for the specific adverse event in a certain subgroup. This figure shows only the adverse events that were identified as CAR-T cell related fatality signals in at least one subgroup. CAR, chimeric antigen receptor; Axi-cel, Axicabtagene ciloleucel; Liso-cel, Lisocabtagene maraleucel; Tisa-cel, Tisagenlecleucel; Brexu-cel, Brexucabtagene autoleucel; Ide-cel, Idecabtagene vicleucel; Cilta-cel, Ciltacabtagene autoleucel; BCMA, B-cell maturation antigen; ICANS, immune effector cell-associated neurotoxicity syndrome; DLC, depressed level of consciousness; IPI, intracranial pressure increased; PAH, pulmonary alveolar hemorrhage; ARDS, acute respiratory distress syndrome; DIC, disseminated intravascular coagulation; ACS, abdominal compartment syndrome; HLH, hemophagocytic lymphohistiocytosis.

To further refine the spectrum of prognostically significant AEs in CAR-T-treated patients with different clinical characteristics, we performed stratified analyses based on age, sex, CAR-T cell drug, and cancer type. Several key patterns in the prognostic impact of CAR-T cell-related AEs across different subgroups were revealed (Fig. 5; Supplementary Figures S4 and S5). First, some AEs showed prognostic significance in almost all subgroups. For example, HLH, disseminated intravascular coagulation, and infectious complications were associated with high-fatality outcomes; while CRS, headache, and hypoalbuminemia were associated with low-fatality outcomes. Second, certain AEs were prognostically significant only in specific subgroups. For instance, Bell's palsy, facial paralysis, and somnolence were associated with low fatality risk in male, but not female. Third, a few AEs displayed diametrically opposed prognostic implications across subgroups, including ICANS, encephalopathy, and hypogammaglobulinemia. ICANS was associated with better patient outcomes among patients receiving anti-CD19 CAR-T cell therapies, but worse outcomes in those treated with anti-BCMA CAR-T cell therapies. Encephalopathy followed a similar trend. Additionally, the prognostic impact of hypogammaglobulinemia varied with age.

**Fig. 6:**
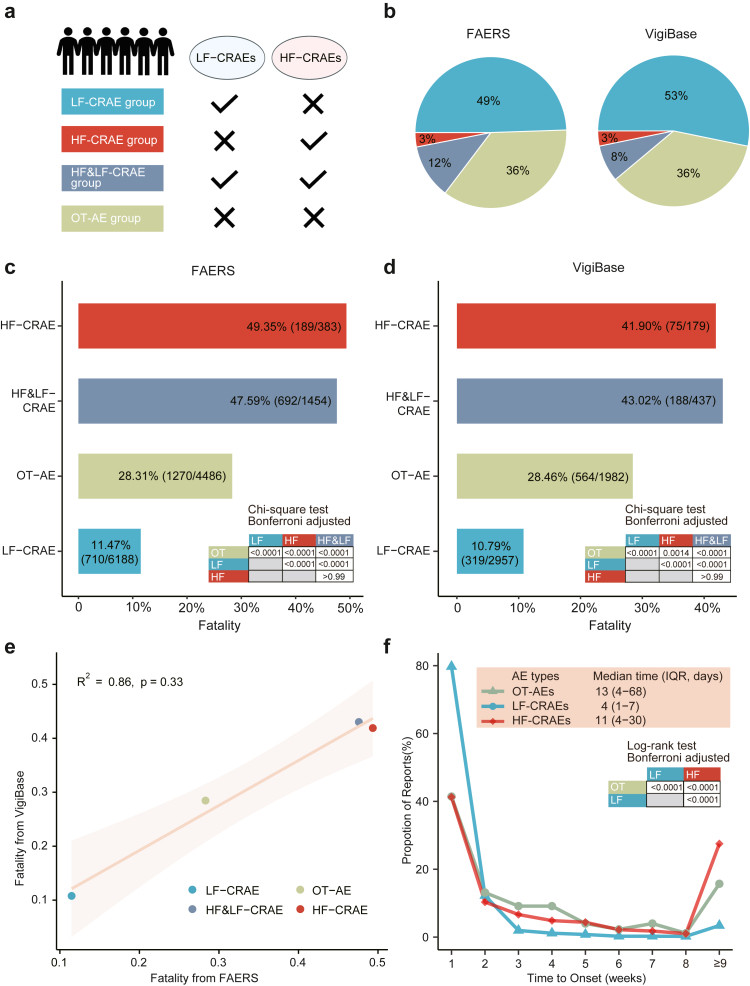
**Fatality analysis of patient clusters according to adverse event types**. (**a**) Schematic of patient grouping based on adverse event profiles (**b**) Proportion distribution of different patient clusters in the FAERS and VigiBase databases. (**c, d**) Comparison of fatality rates across different patient clusters in FAERS and VigiBase, presented as the numbers of fatal cases/total numbers of cases. The Chi-square test with Bonferroni adjustment was used to evaluate statistical significance. (**e**) Correlation of fatality rates across patient clusters between VigiBase and FAERS. A weighted Spearman correlation analysis was performed, with the number of patients in each cluster used as the weighting factor. (**f**) Time-to-onset analysis for each adverse event type based on the VigiBase data. The onset trend is shown in weekly intervals over the first 8 weeks following treatment initiation. Statistical significance was assessed using the Log-rank test with Bonferroni adjustment. CAR, chimeric antigen receptor; HF-CRAEs, high-fatality CAR-T therapy related adverse events; LF-CRAEs, low-fatality CAR-T therapy related adverse events; HF&LF-CRAEs, concurrent high-fatality and low-fatality CAR-T therapy related adverse events; OT-AEs, other adverse events.

Next, CAR-T cell treated cases were categorized into four groups based on their AE profiles: high-fatality CAR-T therapy related AE (HF-CRAE), low-fatality CAR-T therapy related AE (LF-CRAE), concurrent high-fatality and low-fatality CAR-T therapy related AE (HF&LF-CRAE), and OT-AE groups (Fig. 6a). Those who experienced high fatal adverse events were 14.68% (1837/12,511) and 11.09% (616/5555) in FAERS and VigiBase, respectively (Fig. 6b). The HF-CRAE and HF&LF-CRAE groups showed similar fatality rates of 49.35% (189/383) and 47.59% (692/1454), respectively. The LF-CRAE and OT-AE groups showed low and moderate fatality rates of 11.47% (710/6188) and 28.31% (1270/4486), respectively (Fig. 6c). Consistent findings were observed in the VigiBase database. The fatality rates for the HF-CRAE and HF&LF-CRAE groups were 41.90% (75/179) and 43.02% (188/437), respectively, while the LF-CRAE and OT-AE groups showed fatality rates of 10.79% (319/2957) and 28.46% (564/1982), respectively (Fig. 6d). Results from the Chi-square test with Bonferroni adjustment indicated no significant difference between HF-CRAE and HF&LF-CRAE (p > 0.99), while outcomes differed significantly among the other AE clusters (p < 0.0001). The correlation analysis between RORs from FAERS and VigiBase had a *R*^2^ of 0.86 (Fig. 6e). Multivariable Cox regression analyses further supported these observations. Compared with the OT-AE group, the HF-CRAE and HF&LF-CRAE groups showed elevated fatality risks, with adjusted HRs of 2.25 (95% CI: 1.75–2.90) and 1.92 (95% CI: 1.65–2.23), respectively. As expected, the LF-CRAE group had a significantly lower fatality rate (adjusted HR: 0.41; 95% CI: 0.35–0.48). The proportion distribution of prognostically significant high- and low-fatality AE profiles in VigiBase were generally consistent with those observed in FAERS (Supplementary Fig. S6a and b).

Time-to-onset analysis showed that LF-CRAEs tended to occur early after treatment. 79.74% (2480/3110) of LF-CRAEs occurred within the first week, with a median onset time of 4 (interquartile range [IQR]: 1–7) days. This early onset was primarily driven by acute toxicities including CRS and ICANS. In contrast, the median time to onset for HF-CRAEs and OT-AEs was 11 (IQR: 4–30) and 13 (IQR: 4–68) days, respectively (Fig. 6f).

Considering that the incidence of the same AEs varies across different CAR-T products, we summarized the reporting proportions for CRS and ICANS in the FAERS, which are both relatively specific and common AEs in CAR-T therapy. These proportions were compared with incidence from clinical studies to assess whether the reporting patterns in the pharmacovigilance database generally align with those observed in clinical research (Supplementary Table S11). Due to treatment allocation biases and commercial interests, there is currently a lack of appropriate head-to-head comparisons of safety between different CAR-T products. Additionally, the reported incidence of AEs for the same product can vary greatly between real-world studies. Therefore, we did not use quantitative statistical methods to compare the AE reporting proportions in the pharmacovigilance database with the incidence observed in clinical studies. However, there is reliable evidence suggesting that axi-cel has a higher incidence of CRS (86.12% [180/209] vs. 75.60% [158/209]) and ICANS (48.80% [102/209] vs. 22.01% [46/209]) compared to tisa-cel, based on propensity score matching by Bachy et al.^24^ A similar trend was observed in FAERS, where axi-cel had higher reporting proportions for CRS (52.48% [3106/5919] vs. 42.62% [1302/3055]) and ICANS (41.98% [2485/5919] vs. 21.64% [661/3055]) compared to tisa-cel. However, we found that the reported proportion of CRS was lower than the clinical incidence, with a notable example being cilta-cel, which had a CRS reporting proportion of 17.90% in FAERS compared to 70–80% in clinical studies.^25^^,^^26^ This discrepancy may reflect underreporting of common and expected AEs.

## Discussion

**Fig. 7:**
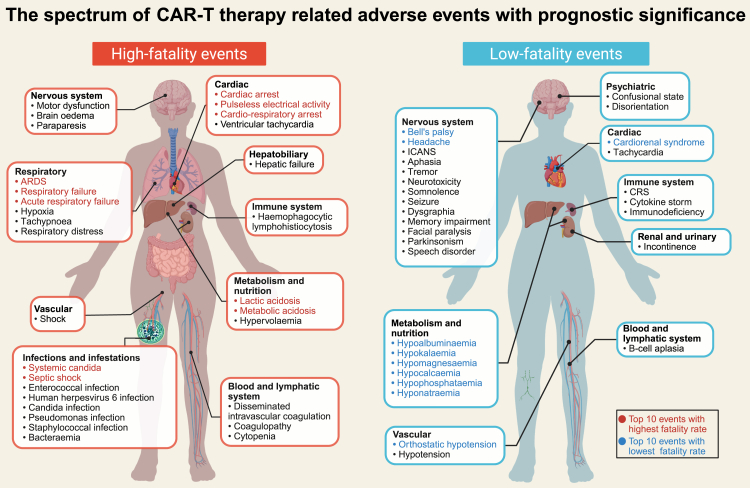
**The spectrum of high-fatality and low-fatality CAR-T therapy related adverse events by affected organs.** Preferred terms in the top 30 occurrences of each group are displayed. CAR, chimeric antigen receptor; ICANS, immune effector cell-associated neurotoxicity syndrome; CRS, cytokine release syndrome; ARDS, acute respiratory distress syndrome.

Treatment-related AEs occur in nearly all patients undergoing CAR-T cell administration, with a broad spectrum of toxicity. Utilizing real-world pharmacovigilance data, this study systematically investigated the prognostic significance of individual AEs in CAR-T cell treated patients. We identified 59 AEs associated with high fatality rates, which occurred in 10%–15% of reported cases. Among these, cardiac events and respiratory/infection-related complications showed notably high fatality and high reporting frequencies, respectively, which is consistent with previous reports highlighting significant non-relapse deaths induced by them in CAR-T cell therapy.^7^ Moreover, we identified 31 AEs with disproportionately reduced fatality rates, which may serve as potential indicators of effective CAR-T cell therapy. Finally, we delineate a spectrum of prognostically significant AEs in CAR-T cell therapy (Fig. 7). These findings provide a resource to better understand the impact of various AEs on patient outcomes and to inform future research into personalized management and the optimization of CAR-T cell based on toxicity patterns.

The relationship between the toxicity and efficacy of CAR-T cell therapy has received limited attention. Most studies focused on the two prominent toxicities, CRS and ICANS, which are closely related to the in vivo expansion of CAR-T cells.^4^, ^5^, ^6^ There is general concern about a lack of CAR-T cell expansion and response in patients without CRS. However, clinical evidence regarding the impact of CRS and ICANS on patient outcomes is contradictory. Some studies showed that non-severe CRS is associated with better patient outcomes,^8^^,^^10^ while others reported non-significant differences.^9^ This may be attributed to heterogeneity in study designs and patients. Our analysis using extensive real-world data indicated that both CRS and ICANS have disproportionately reduced fatal outcomes compared to other AEs. While we cannot completely rule out reporting bias, several observations could counter this explanation. Most notably, ICANS showed opposing prognostic implications in anti-CD19 vs. anti-BCMA CAR-T therapies. This discrepancy is unlikely to be attributable to reporting patterns alone, as there is no evidence that documentation practices differ fundamentally by target antigen. Furthermore, a similar trend was also observed with the related term encephalopathy. However, due to the lack of grading data in pharmacovigilance databases, the observed protective associations likely reflect the effects of non-severe (grade 1–2) CRS and ICANS, which are more frequently encountered in clinical practice.^24^ In contrast, severe CRS frequently co-occurs with hypoxic complications or cardiotoxicity, which were identified as high-fatality events in our study. Given that outcomes aligned with the high-fatality category when high- and low-fatality events are reported simultaneously, a comprehensive view is necessary. Compared to conventional anticancer treatments, CAR-T cell therapy is a high-cost approach. Although the approved indications are expanding, access to this treatment is available to a small proportion of eligible patients worldwide. Thus, most clinical studies can only collect limited sample sizes that are insufficient to support investigation of the prognostic significance of most AEs. Several studies have explored the prognostic value of the comorbidity index for CAR-T cell therapy, but without targeting specific AEs.^27^^,^^28^

Compared to previous pharmacovigilance studies,^13^, ^14^, ^15^, ^16^ the unique contribution of this study is its two-stage analytic framework, which builds on conventional treatment-related safety signals and further identifies signals related to patient outcomes. This approach moves the focus of pharmacovigilance beyond detecting treatment-related events, toward understanding the potential direction and magnitude of the impact of AEs on patient outcomes. However, our proposed spectrum of prognostically significant CAR-T cell therapy-related AEs must be cautiously interpreted, as we cannot determine the specific causes of death. A recent meta-analysis indicated that non-relapse mortality in CAR-T cell therapy ranged between 5.7 and 10.6% across different disease entities.^7^ In contrast, the average fatality rate for CAR-T cell-treated cases in the FAERS database was 22.9%, which is significantly higher than the 8.8% fatality rate in the overall FAERS database, consistent with the poor prognosis of malignancies. This suggests that the majority of deaths reported for CAR-T cell treated cases are likely attributable to cancer-related causes. For instance, high-fatality AE lactic acidosis may indicate cancer progression.^29^ Thus, these prognostically significant AEs affected all-cause mortality in patients, potentially by directly leading to death, interacting with CAR-T cells, and/or affecting disease progression.

When interpreting the clinical relevance of our findings, it is important to consider several inherent features of pharmacovigilance databases. The study population consists of cases for which safety reports were submitted, not all patients receiving CAR-T cell therapy. Therefore, our analysis reflects reporting frequencies/proportions rather than incidence. Mild or expected AEs are likely underreported, as illustrated by the lower reporting rates of CRS and cytopenias compared to their incidence in clinical trials. In contrast, severe AEs, such as HLH, are more likely to be reported. In the safety signal detection, reporting bias may lead to an overrepresentation of severe events, while common or mild adverse events might be underestimated. For prognostically significant signal detection, acute events causing rapid death, such as cardiac arrest, are probably documented more reliably, while atypically presented AEs, including occult infections, may be underreported or delayed in reporting. Thus, the signals identified in this study should be understood as statistical associations within the spontaneous reporting system, rather than as definitive causal relationships. Additionally, analysis at the MedDRA preferred term level enables precise signal detection and provides specific clinical clues, but it also introduces some redundancy. Some independent preferred terms, such as HLH, cytopenia, and thrombocytopenia, may reflect the same clinical event. The identification of these independent terms as fatal signals further strengthens the evidence that HLH and related bone marrow suppression constitute serious complications in CAR-T cell therapy. Interestingly, we found that CRS represents a signal of reduced mortality, while hypoxia, often seen in CRS, points to higher mortality. This suggests that hypoxia may reflect more severe CRS or additional pulmonary complications, which highlight the importance of considering clinical severity when interpreting signals. Whether mild cardiac or infectious events also signal an unfavorable progression trajectory warrants further exploration.

Although causality cannot be established, the identification of prognostic signals provides insights into questions that clinical trials are often unable to answer. Using a two-phase analytic framework, this study identified events that are not only associated with CAR-T therapy but also correlated with patient outcomes. While many high-fatality events reconfirm established risks, the additional value of this work includes quantifying these associations against a large-scale comparator set. Importantly, because these events were first filtered through CAR-T specific safety signals rather than reflecting background events common in hematologic malignancies, the findings underscore safety concerns that are relatively specific to CAR-T cell therapy and therefore provide a useful reference for designing future trials and optimizing CAR-T constructs. An intriguing observation is that ICANS showed opposite prognostic implications in anti-CD19 and anti-BCMA CAR-T therapies, suggesting that differences in antigen target, construct design, and baseline disease context may collectively shape toxicity-outcome relationships. We also observed variations in prognostically significant AEs across different sex and age groups. Furthermore, the prognostic spectrum also generated new hypotheses of clinical relevance, such as hypocalcemia being associated with improved outcomes, whereas hypercalcemia correlated with higher fatality. However, hypercalcemia is not a safety signal specific to CAR-T therapy and therefore may reflect cancer-related conditions. Overall, by constructing a comprehensive landscape of prognostically significant AEs, this study provides a data-driven resource that can help set priorities for monitoring, endpoint selection, and sample size estimation in future toxicity management studies, including product- or indication-specific investigations. These findings also offer real-world evidence to support the integration of AE profiles with established prognostic factors such as Eastern Cooperative Oncology Group performance status, LDH levels, T-cell composition, and tumor burden.^30^

Several limitations of this study must be acknowledged. First, the death causes and specific time-points were not available; therefore, as discussed previously, fatality should be interpreted as all-cause rather than causality with AEs. Temporal ambiguities regarding the onset and outcomes of AEs hinder precise assessment of event sequences, while limited longitudinal follow-up constrains insight into long-term prognostic implications. To enhance the specificity of the signals to CAR-T therapy, we first identified AEs associated with CAR-T and then analyzed their impact on outcomes. Additionally, through multivariable Cox regression accounting for time between treatment initiation and AE reporting, we found that distinct clusters of AEs remained significantly associated with outcomes. However, for individual AEs, the influence of early vs. late onset on short- and long-term patient outcomes remains unclear. Second, the self-reported nature of AE coding using MedDRA introduces potential reporting bias and variability, and the specific diagnostic criteria were unclear. Nevertheless, disproportionality analyses based on spontaneous reporting surveillance databases have proven valuable for enabling large-scale safety analyses of CAR-T cell therapy.^13^, ^14^, ^15^, ^16^^,^^22^ Additionally, 80% of CAR-T cell safety reports were submitted by healthcare professionals, suggesting that general medical practices should be largely followed. Third, due to the lack of information regarding severity, we assessed the relationship between AEs of any grade and patient outcomes. The prognostic impacts observed likely reflect the most frequently encountered severity levels in clinical practice; for example, the classification of CRS as a low-fatality event may be driven mainly by grade 1–2 cases.^24^ Further studies incorporating severity assessments are needed to refine prognostic interpretations. Fourth, AEs may be influenced by concomitant medications; for instance, hypotension could be induced by adrenal insufficiency during steroid taper. We attempted to mitigate this by restricting analysis to reports where CAR-T was the primary suspected agent and by focusing on AEs identified as CAR-T-specific signals. Finally, the available clinical information was limited. Although we adjusted for potential confounders through multivariable regression analysis to verify findings, several important patient-level variables such as Eastern Cooperative Oncology Group performance status, LDH, prior therapies, disease stage, and toxicity management regimen were not included. Additionally, despite the global nature of the data, race and ethnicity information was not available, limiting our ability to assess the population representativeness of these findings. Given these limitations, the current analysis is exploratory, revealing general patterns regarding the prognostic significance of AEs in real-world settings. These safety signals require validation, ideally in prospective studies.

In conclusion, this study systematically evaluated AEs with prognostic implications in CAR-T cell therapy, creating a data-driven resource to inform monitoring strategies and endpoint selection in future studies. Further studies are warranted to investigate whether interventions for high-fatality events can reverse unfavorable clinical outcomes.

## Contributors

Concept and design: HJ., ZS., and GC.

Acquisition, investigation, or interpretation of data: HJ., ZS., JG., HW., XL., JL., ZL., and HZ.

Drafting of the manuscript: ZS., JG., HW., SW., and WS.

Critical review of the manuscript for important intellectual content: All authors.

Formal analysis: ZS., ML., and JG.

Validation: HW. and ZL.

Visualization: JG., ZS., ZY., YO., and YZ.

Supervision: HJ., GC, and SX.

All authors had full access to all the data in the study and accept responsibility for the submission of this manuscript for publication. ZS., HJ., JG., HW., and ML. directly accessed and verified the underlying data reported in this work.

All authors read and approved the final version of the manuscript.

## Data sharing statement

Access to case-level data in the VigiBase is restricted, and researchers can submit an application to the Uppsala Monitoring Centre and obtain license from the Uppsala Monitoring Centre's Approve Committee to access the VigiBase database (https://who-umc.org/). The raw data from the FDA Adverse Event Reporting System are publicly available and can be accessed at: https://www.fda.gov/drugs/drug-approvals-and-databases/fda-adverse-event-reporting-system-faers-database. All summary-level results and data not presented in the manuscript or Supplementary Material will be available with publication upon reasonable request to the corresponding author.

## Editor note

The Lancet Group takes a neutral position with respect to territorial claims in published maps and institutional affiliations.

## Declaration of interests

All authors declare no competing interests.
