# Supplementary material for: Prognostic implications of adverse events associated with CAR-T cell therapy: a population-based global observational study
Source: eClinicalMedicine. 2025 Nov 3;90:103623. doi: 10.1016/j.eclinm.2025.103623 (PMC12766492; doi:10.1016/j.eclinm.2025.103623)
Supplement: Supplementary Figures and Tables [file mmc1.pdf]

Prognostic implications of adverse events associated with CAR-T cell therapy: a population-based global observational study

Supplementary Figure 1. Identification of High-fatality and Low-fatality CAR-T Therapy Related Adverse Events. 2
Supplementary Figure 2. Sankey Plot for the MedDRA Hierarchical Relationship of High-fatality CAR-T therapy related Adverse Events........ 3
Supplementary Figure 3. Sankey Plot for the MedDRA Hierarchical Relationship of Low-fatality CAR-T Therapy related Adverse Events. 4
Supplementary Figure 4. Distribution of High-fatality CAR-T Therapy Related Signals at the System Organ level Across Different Subpopulation. 5
Supplementary Figure 5. Distribution of Low-fatality CAR-T Therapy Related Signals at the System Organ level Across Different Subpopulation. 6
Supplementary Figure 6. Distribution and Organ System Involvement of Prognostically Significant CAR-T Therapy related Adverse Events in VigiBase. 7
Supplementary Table 1. Included System Organ Class Structured by MedDRA (version 26.1) 8
Supplementary Table 2. The Drug-adverse Event Contingency Table Used for Identifying CAR-T cell Therapy Safety Signal 9
Supplementary Table 3. The Patient Outcome-adverse Event Contingency Table Used for Identifying Fatality-related Adverse Events 10
Supplementary Table 4. Clinical Characteristics of Reports of Adverse Events Secondary to CAR-T cell Therapy 11
Supplementary Table 5. Top 30 Most Frequent Drugs in the Non-CAR-T Comparison Group 13
Supplementary Table 6. The 266 CAR-T Therapy Safety Signals Identified by Disproportionality Analysis 14
Supplementary Table 7. The Fatality and its Reporting Odds Ratios for High-fatality Adverse Events in CAR-T cell Therapy 21
Supplementary Table 8. The Fatality and its Reporting Odds Ratios for High-fatality CAR-T Therapy related Adverse Events at the System-organ Level. 25
Supplementary Table 9. The Fatality and its Reporting Odds Ratios for Low-fatality Adverse Events in CAR-T cell Therapy 26
Supplementary Table 10. The Fatality and its Reporting Odds Ratios for Low-fatality CAR-T Therapy related Adverse Events at the System-organ Level. 28
Supplementary Table 11. Reporting proportions of CRS and ICANS according to CAR-T drug type. 29

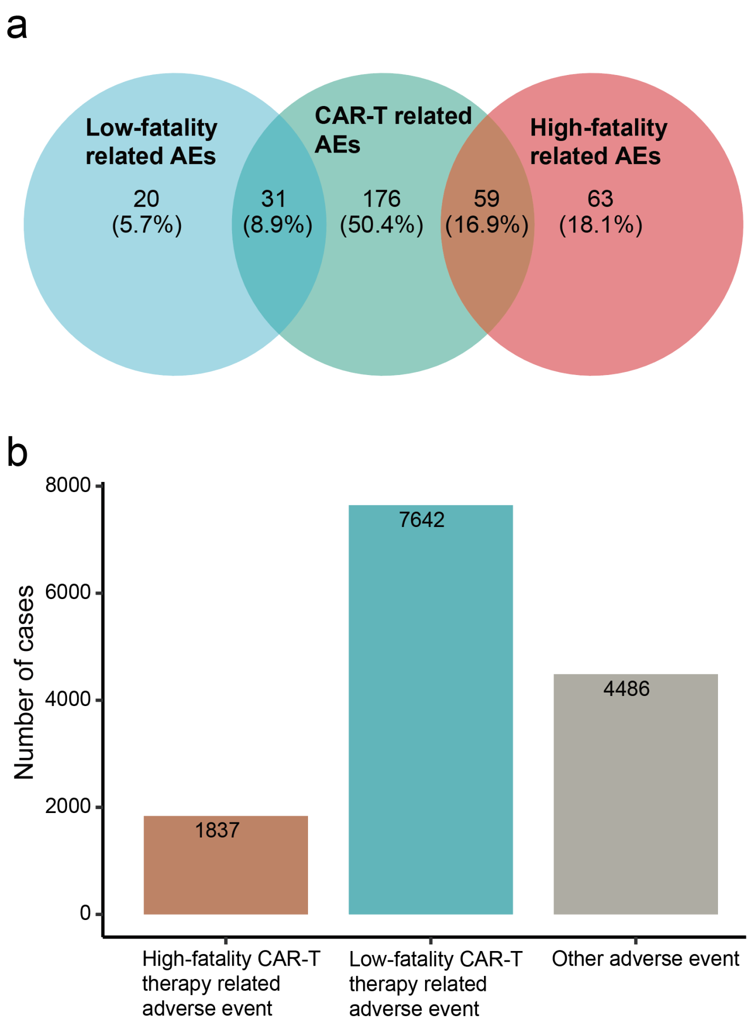

Supplementary Figure 1. Identification of High-fatality and Low-fatality CAR-T Therapy Related Adverse Events.

(**a**) The number of CAR-T therapy related adverse events and fatality-related adverse events identified by disproportionality analysis. (**b**) Distribution of case proportions by the type of adverse event.

CAR, chimeric antigen receptor; AEs, adverse events

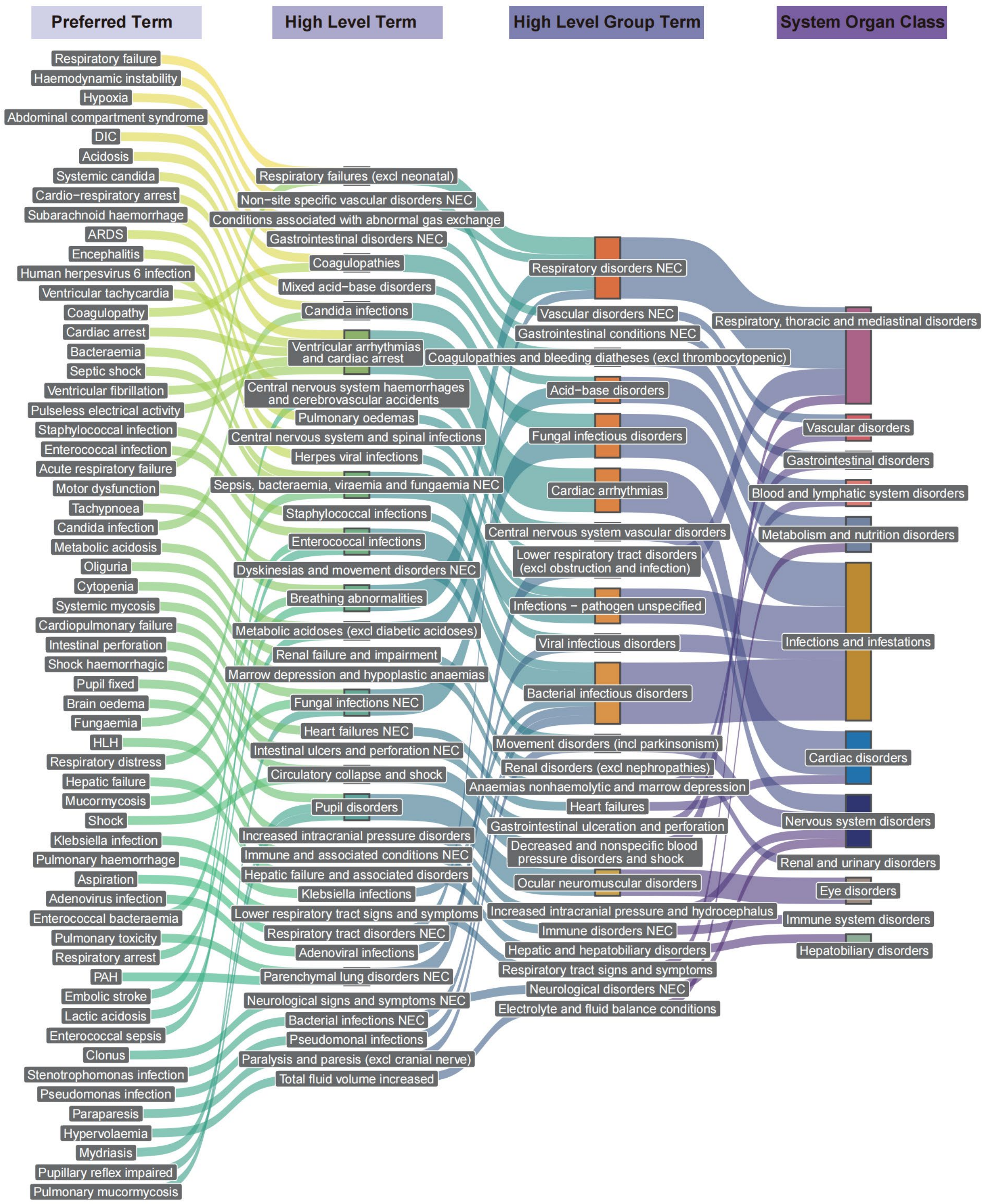

Supplementary Figure 2. Sankey Plot for the MedDRA Hierarchical Relationship of High-fatality CAR-T therapy related Adverse Events. DIC, disseminated intravascular coagulation; ARDS, acute respiratory distress syndrome; HLH, hemophagocytic lymphohistiocytosis; PAH, pulmonary alveolar haemorrhage. NES denotes not classified elsewhere.

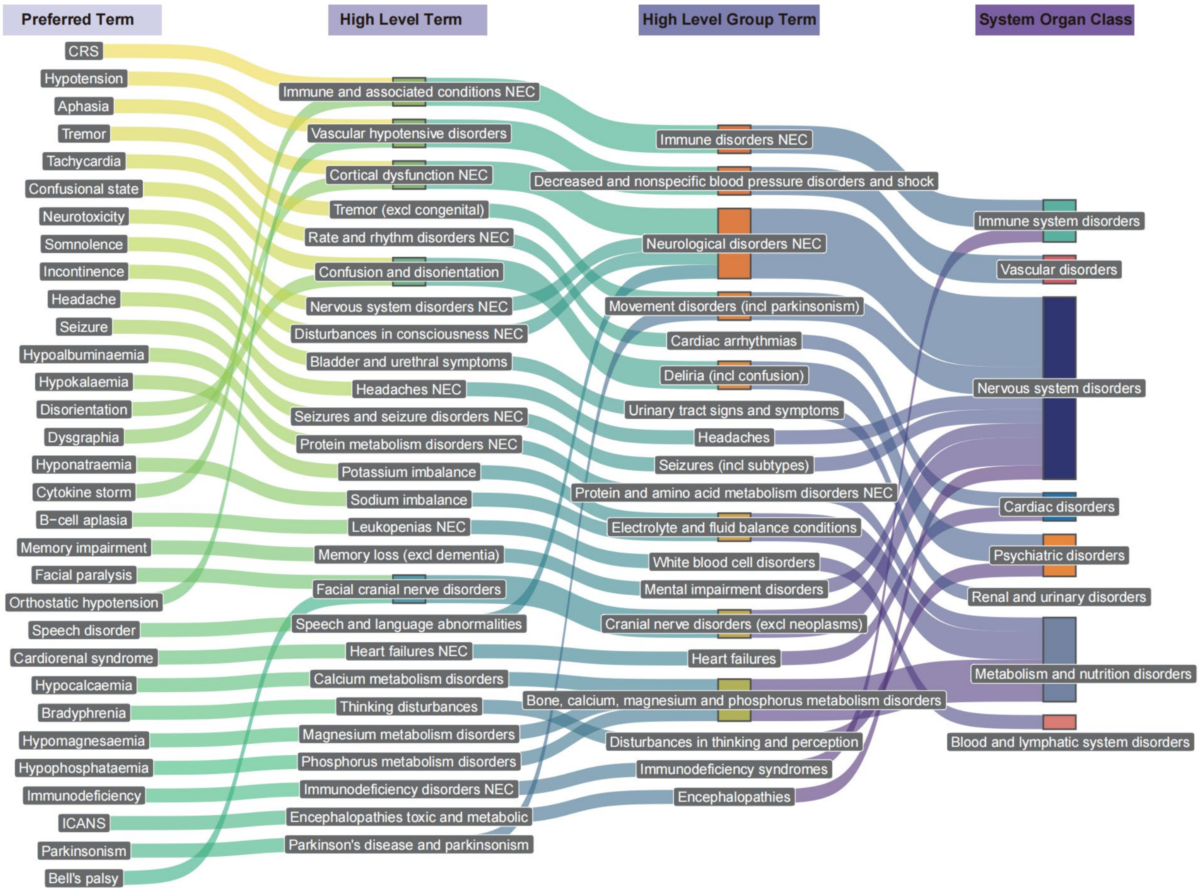

Supplementary Figure 3. Sankey Plot for the MedDRA Hierarchical Relationship of Low-fatality CAR-T Therapy related Adverse Events. CRS, Cytokine release syndrome; ICANS, Immune effector cell-associated neurotoxicity syndrome. NES denotes not classified elsewhere.

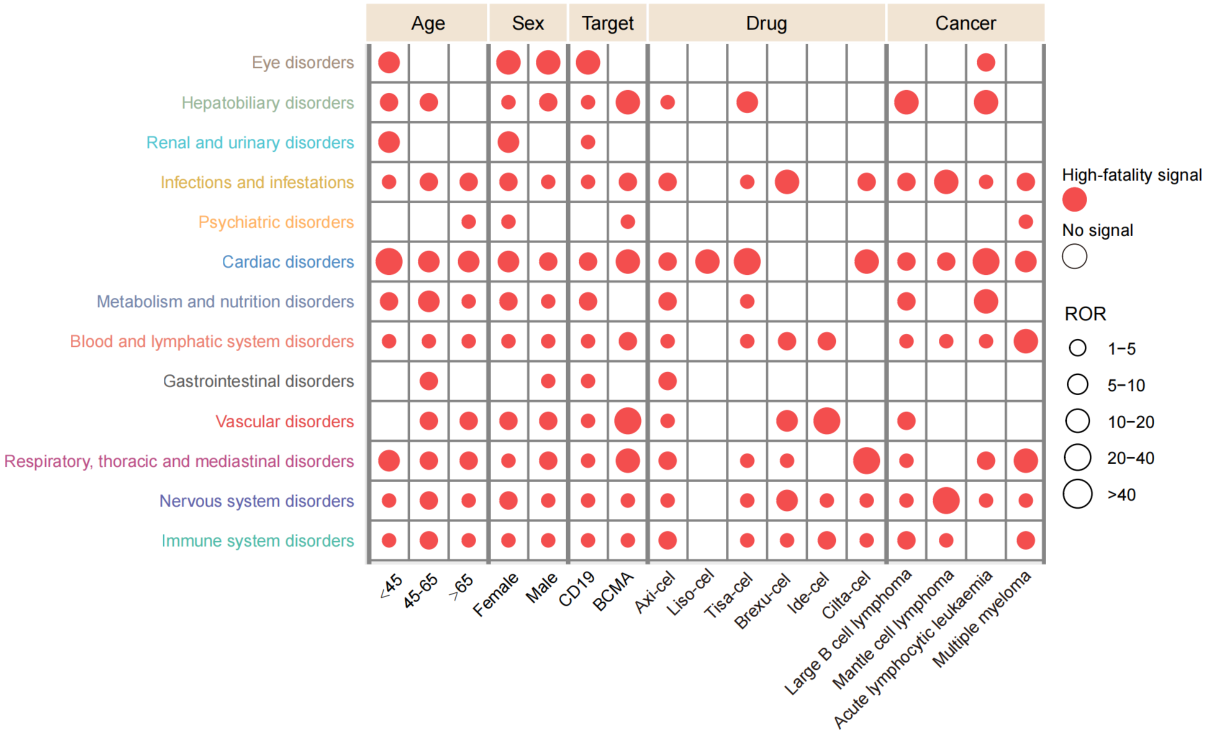

Supplementary Figure 4. Distribution of High-fatality CAR-T Therapy Related Signals at the System Organ level Across Different Subpopulation.

Red squares represent that a subgroup had high-fatality signals belonging to an organ system class. Blank boxes indicate that no death signal belonging to an organ system was detected.

Axi-cel, Axicabtagene ciloleucel; Liso-cel, Lisocabtagene maraleucel; Tisa-cel, Tisagenlecleucel; Brexu-cel, Brexucabtagene autoleucel; Ide-cel, Idecabtagene vicleucel; Cilta-cel, Ciltacabtagene autoleucel; ROR, reporting odds ratios

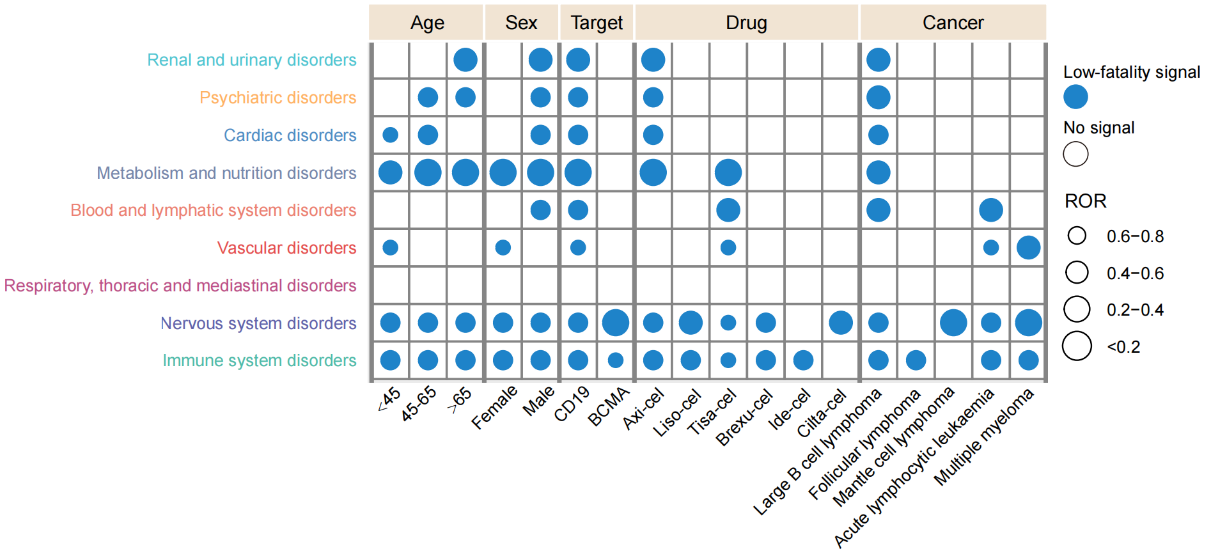

Supplementary Figure 5. Distribution of Low-fatality CAR-T Therapy Related Signals at the System Organ level Across Different Subpopulation.

Blue squares represent that a subgroup had low-fatality signals belonging to an organ system class. Blank boxes indicate that no death signal belonging to an organ system was detected.

Axi-cel, Axicabtagene ciloleucel; Liso-cel, Lisocabtagene maraleucel; Tisa-cel, Tisagenlecleucel; Brexu-cel, Brexucabtagene autoleucel; Ide-cel, Idecabtagene vicleucel; Cilta-cel, Ciltacabtagene autoleucel; ROR, reporting odds ratios

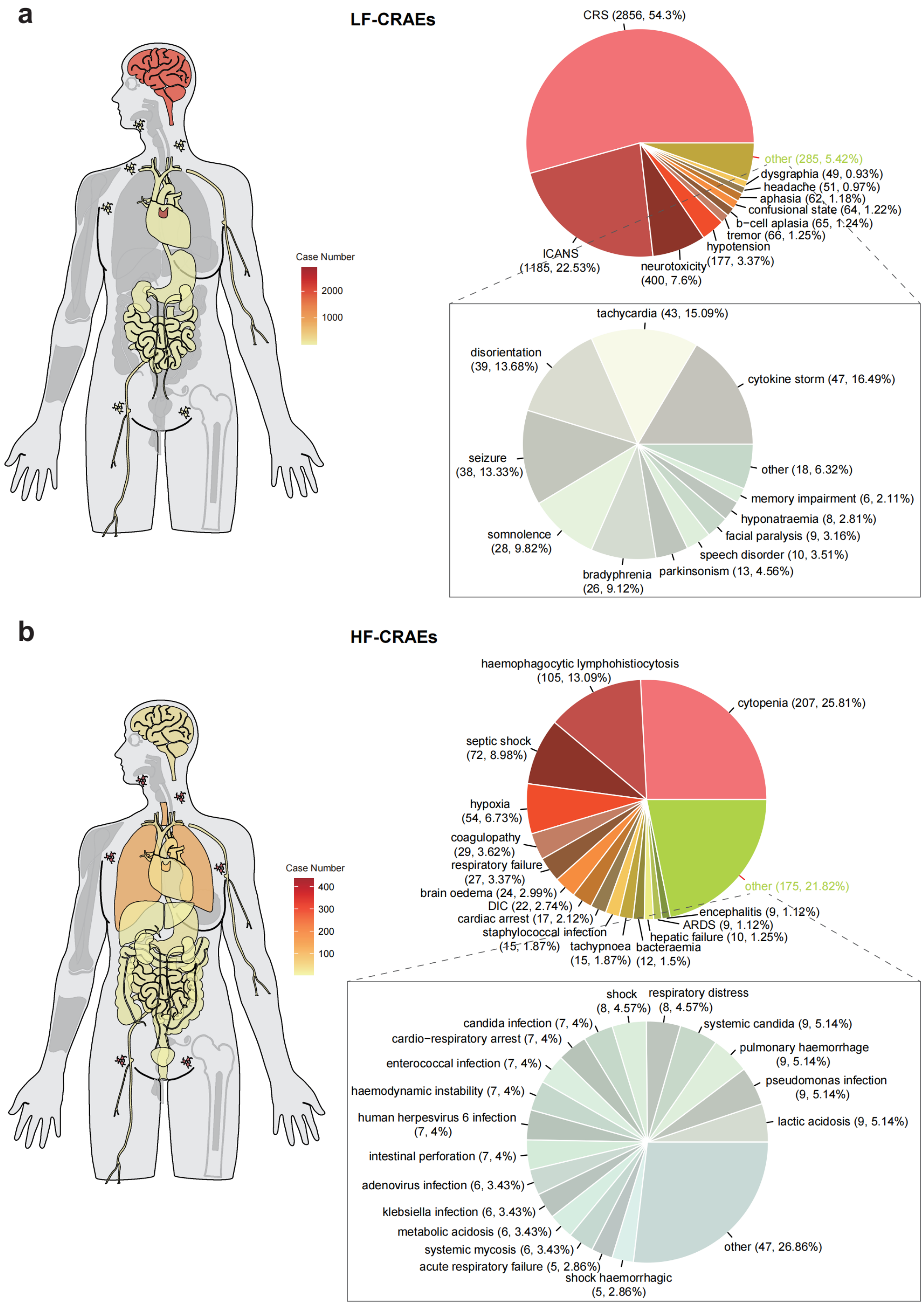

Supplementary Figure 6. Distribution and Organ System Involvement of Prognostically Significant CAR-T Therapy related Adverse Events in VigiBase.

(**a**) Low-fatality CAR-T therapy related adverse events. (**b**) High-fatality CAR-T therapy related adverse events. Human anatomy heatmaps were created with the MOAHIT web tool (v1.0, https://smuonco.shinyapps.io/MOAHIT/).

LF-CRAE, low-fatality CAR-T therapy related adverse event; HF-CRAE, high-fatality CAR-T therapy related adverse event; CRS, cytokine release syndrome; ICANS, immune effector cell-associated neurotoxicity syndrome; DIC, disseminated intravascular coagulation; ARDS, acute respiratory distress syndrome.

Supplementary Table 1. Included System Organ Class Structured by MedDRA (version 26.1)

Blood and lymphatic system disorders
Cardiac disorders
Ear and labyrinth disorders
Endocrine disorders
Eye disorders
Gastrointestinal disorders
Hepatobiliary disorders
Immune system disorders
Infections and infestations
Metabolism and nutrition disorders
Musculoskeletal and connective tissue disorders
Nervous system disorders
Psychiatric disorders
Renal and urinary disorders
Reproductive system and breast disorders
Respiratory, thoracic and mediastinal disorders
Skin and subcutaneous tissue disorders
Vascular disorders

Supplementary Table 2. The Drug-adverse Event Contingency Table Used for Identifying CAR-T cell Therapy Safety Signal

	Target adverse events	All other adverse events	Total
CAR-T cell drugs	a	b	a+b
non-CAR-T drugs*	c	d	c+d
Total	a+c	b+d	a+b+c+d

a: number of reports of target adverse events from CAR-T cell therapy drugs; b: number of reports of other adverse events from CAR-T cell therapy drugs; c: number of reports of target adverse events from non-CAR-T comparator drugs; d: number of reports of other adverse events from non-CAR-T comparator drugs.

* cases with hematologic malignancies (lymphoma, lymphoblastic leukemia, or plasma cell myeloma) in which non-CAR-T therapy is the primary suspected drug.

Supplementary Table 3. The Patient Outcome-adverse Event Contingency Table Used for Identifying Fatality-related Adverse Events

	Target adverse events	All other adverse events	Total
Death outcome	a	b	a+b
Non-death outcome	c	d	c+d
Total	a+c	b+d	a+b+c+d

a: number of cases with the target adverse event in death cases; b: number of cases without the target adverse event in death cases; c: number of cases with the target adverse event in non-death cases; d: number of cases without the target adverse event in non-death cases.

Supplementary Table 4. Clinical Characteristics of Reports of Adverse Events Secondary to CAR-T cell Therapy

Characteristic	**Overall** (N = 12,511)	**Death** (N = 2,861)	**Non-death** (N = 9,650)	p value
**Sex**				0.016
Male	6,064 (48.47%)	1,454 (50.82%)	4,610 (47.77%)	
Female	3,729 (29.81%)	816 (28.52%)	2,913 (30.19%)	
Missing	2,718 (21.72%)	591 (20.66%)	2,127 (22.04%)	
**Age (years)**	61.00 (47.00, 69.00)	61.00 (46.00, 69.00)	62.00 (48.00, 69.00)	0.27
**Drug targets**				<0.0001
CD19	10,489 (83.84%)	2,641 (92.31%)	7,848 (81.33%)	
BCMA	2,022 (16.16%)	220 (7.69%)	1,802 (18.67%)	
**Drug**				<0.0001
Axi-cel	5,919 (47.31%)	1,422 (49.70%)	4,497 (46.60%)	
Tisa-cel	3,055 (24.42%)	881 (30.79%)	2,174 (22.53%)	
Brexu-cel	1,138 (9.10%)	277 (9.68%)	861 (8.92%)	
Cilta-cel	1,363 (10.89%)	158 (5.52%)	1,205 (12.49%)	
Ide-cel	659 (5.27%)	62 (2.17%)	597 (6.19%)	
Liso-cel	377 (3.01%)	61 (2.13%)	316 (3.27%)	
**Cancer type**				
Large B-Cell Lymphoma	4,188 (33.47%)	1,059 (37.02%)	3,129 (32.42%)	<0.0001
Acute lymphocytic leukaemia	1,434 (11.46%)	313 (10.94%)	1,121 (11.62%)	
Multiple myeloma	1,061 (8.48%)	157 (5.49%)	904 (9.37%)	
Mantle cell lymphoma	620 (4.96%)	151 (5.28%)	469 (4.86%)	
Follicular lymphoma	227 (1.81%)	25 (0.87%)	202 (2.09%)	
Chronic lymphocytic leukaemia	26 (0.21%)	8 (0.28%)	18 (0.19%)	
Missing	4,955 (39.61%)	1,148 (40.13%)	3,807 (39.45%)	
**Country**				<0.0001
United States	7,777 (62.16%)	1,378 (48.16%)	6,399 (66.31%)	
France	471 (3.76%)	116 (4.05%)	355 (3.68%)	
Spain	421 (3.37%)	165 (5.77%)	256 (2.65%)	
Germany	372 (2.97%)	158 (5.52%)	214 (2.22%)	
Italy	325 (2.60%)	204 (7.13%)	121 (1.25%)	
United Kingdom	286 (2.29%)	98 (3.43%)	188 (1.95%)	
Japan	237 (1.89%)	95 (3.32%)	142 (1.47%)	
Australia	234 (1.87%)	98 (3.43%)	136 (1.41%)	
China	204 (1.63%)	35 (1.22%)	169 (1.75%)	
Canada	146 (1.17%)	41 (1.43%)	105 (1.09%)	
Other/missing	2,038 (16.29%)	473 (16.53%)	1,565 (16.22%)	
**Reporter**				<0.0001
Health professional	10,009 (80.00%)	2,510 (87.73%)	7,499 (77.71%)	
Consumer	1,616 (12.92%)	260 (9.09%)	1,356 (14.05%)	
Other/missing	886 (7.08%)	91 (3.18%)	795 (8.24%)	
**Event year**				<0.0001
2017	10 (0.08%)	4 (0.14%)	6 (0.06%)	
2018	636 (5.08%)	80 (2.80%)	556 (5.76%)	

2019	1,172 (9.37%)	191 (6.68%)	981 (10.17%)
2020	1,603 (12.81%)	417 (14.58%)	1,186 (12.29%)
2021	1,670 (13.35%)	399 (13.95%)	1,271 (13.17%)
2022	2,247 (17.96%)	530 (18.52%)	1,717 (17.79%)
2023	2,998 (23.96%)	707 (24.71%)	2,291 (23.74%)
2024	2,175 (17.38%)	533 (18.63%)	1,642 (17.02%)

Data are shown as number (percentage) or median (interquartile range). p values were calculated using Pearson's Chi-squared test for categorical variables and Wilcoxon rank sum test for continuous variables.

BCMA, B-cell maturation antigen

Supplementary Table 5. Top 30 Most Frequent Drugs in the Non-CAR-T Comparison Group

Drugs	Count
Lenalidomide	229035
Pomalidomide	65547
Dexamethasone	63496
Ibrutinib	57178
Rituximab	55147
Cyclophosphamide	50472
Vincristine	41511
Doxorubicin	35311
Bortezomib	26612
Venetoclax	24496
Prednisone	24476
Methotrexate	22454
Cytarabine	18947
Daratumumab	18749
Ixazomib	16327
Etoposide	16008
Bendamustine	13410
Carfilzomib	12656
Prednisolone	10766
Obinutuzumab	9415
Pegaspargase	7313
Thalidomide	6676
Cisplatin	6536
Ifosfamide	6098
Melphalan	5815
Mercaptopurine	5422
Polatuzumab Vedotin	5349
Acalabrutinib	5303
Selinexor	5282
Gemcitabine	5197

Supplementary Table 6. The 266 CAR-T Therapy Safety Signals Identified by Disproportionality Analysis

Preferred term	No. of events	ROR	95% CI (LL)	95% CI (UL)	System Organ Class
Disseminated intravascular coagulation	77	4.63	3.64	5.9	Blood and lymphatic system disorders
Bone marrow failure	100	1.5	1.22	1.83	Blood and lymphatic system disorders
Coagulopathy	88	6.66	5.27	8.41	Blood and lymphatic system disorders
Pancytopenia	294	1.79	1.59	2.01	Blood and lymphatic system disorders
B-cell aplasia	96	71.47	48.72	104.83	Blood and lymphatic system disorders
Cytopenia	340	3.69	3.3	4.14	Blood and lymphatic system disorders
Hypofibrinogenaemia	46	3.72	2.73	5.06	Blood and lymphatic system disorders
Coombs negative haemolytic anaemia	4	9.73	3.1	30.55	Blood and lymphatic system disorders
Hypoglobulinaemia	4	3.69	1.3	10.5	Blood and lymphatic system disorders
Erythropenia	3	4.22	1.25	14.27	Blood and lymphatic system disorders
Tachycardia	364	7.83	6.97	8.81	Cardiac disorders
Bradycardia	46	1.91	1.42	2.58	Cardiac disorders
Cardio-respiratory arrest	33	2.25	1.57	3.21	Cardiac disorders
Supraventricular tachycardia	20	2.36	1.49	3.72	Cardiac disorders
Ventricular tachycardia	28	2.71	1.84	4	Cardiac disorders
Sinus tachycardia	33	3.79	2.63	5.46	Cardiac disorders
Cardiac arrest	80	1.66	1.33	2.09	Cardiac disorders
Ventricular fibrillation	12	1.88	1.05	3.37	Cardiac disorders
Pulseless electrical activity	24	10.7	6.67	17.18	Cardiac disorders
Cardiorenal syndrome	46	111.96	57.99	216.16	Cardiac disorders
Cardiopulmonary failure	8	3.57	1.71	7.46	Cardiac disorders
Nodal rhythm	3	20.06	4.49	89.64	Cardiac disorders
Acute left ventricular failure	3	3.34	1.01	11.1	Cardiac disorders
Diabetes insipidus	6	7.64	3.08	18.94	Endocrine disorders
Pupil fixed	5	22.29	6.8	73.04	Eye disorders
Diplopia	21	1.82	1.17	2.84	Eye disorders
Papilloedema	7	2.18	1.01	4.7	Eye disorders
Mydriasis	6	6.98	2.84	17.14	Eye disorders
Pupillary reflex impaired	5	66.87	12.97	344.7	Eye disorders
Anisocoria	9	24.08	9.78	59.25	Eye disorders
Ophthalmoplegia	3	7.29	2.04	26.15	Eye disorders
Abdominal compartment syndrome	5	12.16	4.22	35	Gastrointestinal disorders
Dysphagia	85	1.58	1.27	1.97	Gastrointestinal disorders
Intestinal perforation	20	1.74	1.1	2.73	Gastrointestinal disorders
Small intestinal perforation	7	2.18	1.01	4.7	Gastrointestinal disorders
Pneumoperitoneum	6	4.46	1.88	10.58	Gastrointestinal disorders
Gastric fistula	3	20.06	4.49	89.64	Gastrointestinal disorders
Retroperitoneal haematoma	9	12.67	5.73	28.01	Gastrointestinal disorders
Haemoperitoneum	4	4.12	1.44	11.79	Gastrointestinal disorders
Parotid gland enlargement	3	5.73	1.65	19.95	Gastrointestinal disorders

Immune-mediated enterocolitis	5	4.31	1.68	11.1	Gastrointestinal disorders
Hyperbilirubinaemia	49	2.39	1.79	3.21	Hepatobiliary disorders
Hepatic failure	51	2.2	1.65	2.93	Hepatobiliary disorders
Hepatic necrosis	4	3.57	1.26	10.12	Hepatobiliary disorders
Cytokine release syndrome	5803	55.6	53.18	58.12	Immune system disorders
Cytokine storm	50	38.25	24.83	58.92	Immune system disorders
Hypogammaglobulinaemia	396	10.95	9.74	12.31	Immune system disorders
Haemophagocytic lymphohistiocytosis	262	11.21	9.71	12.96	Immune system disorders
Immunodeficiency	43	1.6	1.17	2.17	Immune system disorders
Cell-mediated immune deficiency	6	20.06	6.96	57.82	Immune system disorders
Graft versus host disease in liver	6	2.77	1.19	6.41	Immune system disorders
Sarcoidosis	6	2.55	1.1	5.89	Immune system disorders
Transplant rejection	3	3.65	1.09	12.19	Immune system disorders
Immune effector cell-associated hlh-like syndrome	6	160.5	19.32	1333.21	Immune system disorders
Systemic candida	28	3.26	2.2	4.82	Infections and infestations
Clostridium difficile colitis	48	1.39	1.04	1.86	Infections and infestations
Encephalitis	17	1.76	1.08	2.88	Infections and infestations
Human herpesvirus 6 infection	27	3.58	2.39	5.34	Infections and infestations
Bacteraemia	75	2.21	1.75	2.8	Infections and infestations
Septic shock	145	1.32	1.11	1.55	Infections and infestations
Staphylococcal infection	73	2.14	1.69	2.72	Infections and infestations
Rhinovirus infection	52	2.82	2.12	3.75	Infections and infestations
Enterococcal infection	53	4.73	3.53	6.33	Infections and infestations
Candida infection	50	1.77	1.33	2.36	Infections and infestations
Systemic mycosis	12	3.53	1.93	6.44	Infections and infestations
Fungaemia	22	6.47	4.06	10.31	Infections and infestations
Pseudomonal bacteraemia	14	3.04	1.75	5.29	Infections and infestations
Viral upper respiratory tract infection	18	1.85	1.15	2.99	Infections and infestations
Lower respiratory tract infection fungal	6	5.18	2.16	12.41	Infections and infestations
Periorbital cellulitis	4	4.86	1.68	14.11	Infections and infestations
Mucormycosis	20	2.19	1.39	3.46	Infections and infestations
Clostridium bacteraemia	4	5.94	2.01	17.56	Infections and infestations
Klebsiella infection	22	2.27	1.47	3.51	Infections and infestations
Meningoencephalitis herpetic	4	2.89	1.03	8.11	Infections and infestations
Gastroenteritis escherichia coli	4	4.12	1.44	11.79	Infections and infestations
Adenovirus infection	24	3.63	2.37	5.56	Infections and infestations
Bk virus infection	28	4.09	2.75	6.09	Infections and infestations
Klebsiella bacteraemia	9	2.87	1.44	5.7	Infections and infestations
Viral sinusitis	17	41.35	19.37	88.28	Infections and infestations
Enterococcal bacteraemia	12	2.92	1.61	5.3	Infections and infestations
Enterococcal sepsis	8	3.19	1.53	6.65	Infections and infestations
Vascular device infection	12	2.53	1.4	4.57	Infections and infestations

Gastrointestinal bacterial infection	4	2.97	1.06	8.35	Infections and infestations
Serratia infection	3	6.17	1.76	21.66	Infections and infestations
Stenotrophomonas infection	10	3.66	1.89	7.1	Infections and infestations
Escherichia bacteraemia	21	1.9	1.22	2.95	Infections and infestations
Corynebacterium infection	6	8.02	3.22	19.98	Infections and infestations
Pseudomonas infection	27	1.67	1.13	2.47	Infections and infestations
Enterovirus infection	8	2.4	1.17	4.96	Infections and infestations
Human herpesvirus 6 encephalitis	17	15.16	8.36	27.49	Infections and infestations
Urinary tract infection enterococcal	5	3.43	1.35	8.7	Infections and infestations
Myelitis	6	2.92	1.26	6.78	Infections and infestations
Cytomegalovirus infection reactivation	33	1.54	1.08	2.18	Infections and infestations
Epstein-barr viraemia	4	3.15	1.12	8.87	Infections and infestations
Enterobacter bacteraemia	3	4.01	1.19	13.5	Infections and infestations
Alpha haemolytic streptococcal infection	9	4.38	2.16	8.86	Infections and infestations
Haemophilus infection	7	3.53	1.61	7.77	Infections and infestations
Adenoviral haemorrhagic cystitis	3	3.49	1.05	11.62	Infections and infestations
Cytomegalovirus viraemia	33	2.1	1.47	2.99	Infections and infestations
Mycobacterium avium complex infection	4	2.89	1.03	8.11	Infections and infestations
Urinary tract infection staphylococcal	4	5.94	2.01	17.56	Infections and infestations
Fusobacterium infection	3	10.03	2.66	37.81	Infections and infestations
Psoas abscess	3	5.73	1.65	19.95	Infections and infestations
Diverticulitis intestinal perforated	3	5.35	1.55	18.48	Infections and infestations
Herpes zoster reactivation	4	3.34	1.18	9.45	Infections and infestations
Pulmonary mucormycosis	5	3.93	1.54	10.06	Infections and infestations
Metapneumovirus pneumonia	3	4.22	1.25	14.27	Infections and infestations
Acidosis	19	5.91	3.6	9.72	Metabolism and nutrition disorders
Malnutrition	37	3.28	2.33	4.61	Metabolism and nutrition disorders
Hypernatraemia	18	4.3	2.61	7.07	Metabolism and nutrition disorders
Hypoalbuminaemia	148	10.31	8.53	12.47	Metabolism and nutrition disorders
Hypokalaemia	107	1.88	1.55	2.29	Metabolism and nutrition disorders
Hyponatraemia	85	1.86	1.49	2.31	Metabolism and nutrition disorders
Metabolic acidosis	31	3.17	2.18	4.59	Metabolism and nutrition disorders
Hypocalcaemia	58	2.23	1.7	2.91	Metabolism and nutrition disorders
Hypomagnesaemia	51	4.49	3.34	6.04	Metabolism and nutrition disorders
Iron overload	3	4.01	1.19	13.5	Metabolism and nutrition disorders
Hypophosphataemia	66	5.32	4.09	6.93	Metabolism and nutrition disorders
Lactic acidosis	37	4.16	2.94	5.88	Metabolism and nutrition disorders
Hyperchloraemia	3	16.05	3.84	67.16	Metabolism and nutrition disorders
Hypervolaemia	64	4.97	3.8	6.49	Metabolism and nutrition disorders
Hypoproteinaemia	12	3.73	2.04	6.83	Metabolism and nutrition disorders
Rhabdomyolysis	9	2.02	1.03	3.98	Musculoskeletal and connective tissue disorders

Compartment syndrome	3	4.72	1.38	16.11	Musculoskeletal and connective tissue disorders
Muscle rigidity	4	5.63	1.92	16.55	Musculoskeletal and connective tissue disorders
Haematoma muscle	3	3.65	1.09	12.19	Musculoskeletal and connective tissue disorders
Status epilepticus	46	8.15	5.86	11.35	Nervous system disorders
Aphasia	336	16.6	14.48	19.02	Nervous system disorders
Encephalopathy	387	12.84	11.37	14.5	Nervous system disorders
Tremor	361	2.92	2.62	3.26	Nervous system disorders
Neurological symptom	46	5.42	3.95	7.45	Nervous system disorders
Nervous system disorder	94	3.93	3.17	4.89	Nervous system disorders
Neurotoxicity	1907	37.06	34.58	39.71	Nervous system disorders
Brain stem infarction	3	8.92	2.41	32.94	Nervous system disorders
Subarachnoid haemorrhage	12	1.81	1.01	3.25	Nervous system disorders
Dysmetria	5	6.37	2.4	16.89	Nervous system disorders
Somnolence	259	2.85	2.5	3.24	Nervous system disorders
Headache	410	1.74	1.58	1.93	Nervous system disorders
Seizure	181	2.74	2.35	3.2	Nervous system disorders
Cerebellar infarction	11	14.71	7.05	30.71	Nervous system disorders
Dysgraphia	93	37.2	27.17	50.93	Nervous system disorders
Generalised tonic-clonic seizure	24	2.95	1.93	4.49	Nervous system disorders
Spinal cord oedema	3	10.03	2.66	37.81	Nervous system disorders
Central nervous system lesion	14	2.37	1.37	4.09	Nervous system disorders
Neurological decompensation	15	5.21	3	9.06	Nervous system disorders
Hemiparesis	42	3.78	2.74	5.23	Nervous system disorders
Memory impairment	140	1.48	1.25	1.76	Nervous system disorders
Motor dysfunction	34	7.71	5.27	11.29	Nervous system disorders
Dysarthria	48	2.89	2.14	3.89	Nervous system disorders
Facial paralysis	67	6.17	4.73	8.04	Nervous system disorders
Speech disorder	46	2.4	1.77	3.25	Nervous system disorders
Toxic encephalopathy	14	3.6	2.06	6.29	Nervous system disorders
Cognitive disorder	55	1.51	1.15	1.98	Nervous system disorders
Disturbance in attention	29	1.97	1.35	2.88	Nervous system disorders
Depressed level of consciousness	162	13.42	11.11	16.2	Nervous system disorders
Brain oedema	91	14.26	11.05	18.39	Nervous system disorders
Intracranial pressure increased	15	3.82	2.22	6.57	Nervous system disorders
Slow speech	9	7.77	3.7	16.31	Nervous system disorders
Dyscalculia	7	401.25	22.92	7025.93	Nervous system disorders
Areflexia	4	3.15	1.12	8.87	Nervous system disorders
Unresponsive to stimuli	55	8.18	6.05	11.07	Nervous system disorders
Apraxia	6	3.41	1.46	7.99	Nervous system disorders
Essential tremor	4	5.94	2.01	17.56	Nervous system disorders

Intraventricular haemorrhage	3	6.17	1.76	21.66	Nervous system disorders
Nystagmus	5	4.18	1.63	10.73	Nervous system disorders
Intention tremor	4	10.7	3.36	34.12	Nervous system disorders
Lethargy	63	1.86	1.44	2.4	Nervous system disorders
Mental impairment	18	2.4	1.48	3.88	Nervous system disorders
Coma	27	1.89	1.28	2.79	Nervous system disorders
Cerebral atrophy	9	6.02	2.92	12.4	Nervous system disorders
Cerebral ventricle dilatation	3	26.75	5.4	132.53	Nervous system disorders
Slow response to stimuli	10	22.29	9.63	51.6	Nervous system disorders
Myoclonus	13	4.24	2.36	7.61	Nervous system disorders
Language disorder	8	11.89	5.17	27.34	Nervous system disorders
Optic neuritis	6	4.01	1.7	9.46	Nervous system disorders
Cranial nerve paralysis	15	50.16	21.27	118.33	Nervous system disorders
Noninfective encephalitis	6	22.93	7.71	68.23	Nervous system disorders
Fine motor skill dysfunction	5	5.35	2.05	13.98	Nervous system disorders
Cerebral disorder	13	2.5	1.42	4.42	Nervous system disorders
Cerebellar syndrome	8	2.82	1.36	5.83	Nervous system disorders
Hyporesponsive to stimuli	3	4.46	1.31	15.13	Nervous system disorders
Seizure like phenomena	5	11.15	3.93	31.64	Nervous system disorders
Embolic stroke	7	4.26	1.92	9.45	Nervous system disorders
Facial paresis	13	5.27	2.91	9.55	Nervous system disorders
Myelitis transverse	4	3.82	1.34	10.89	Nervous system disorders
Clonus	7	12.48	5.09	30.62	Nervous system disorders
Altered state of consciousness	37	2.83	2.02	3.97	Nervous system disorders
Dyskinesia	15	2.46	1.45	4.18	Nervous system disorders
Immune effector cell-associated neurotoxicity syndrome	2223	108.22	98.69	118.66	Nervous system disorders
White matter lesion	6	3.34	1.43	7.81	Nervous system disorders
Epilepsy	21	1.6	1.03	2.49	Nervous system disorders
Autoimmune encephalopathy	3	80.24	8.35	771.47	Nervous system disorders
Hyperreflexia	3	6.69	1.89	23.7	Nervous system disorders
Paraparesis	25	13.94	8.59	22.6	Nervous system disorders
Iiird nerve disorder	3	26.75	5.4	132.53	Nervous system disorders
Parkinsonism	57	58.71	36.91	93.36	Nervous system disorders
Intensive care unit acquired weakness	3	6.69	1.89	23.7	Nervous system disorders
Sedation	5	3.34	1.32	8.47	Nervous system disorders
Immune-mediated encephalopathy	3	80.24	8.35	771.47	Nervous system disorders
Hydrocephalus	8	3.51	1.68	7.33	Nervous system disorders
Cerebral mass effect	6	17.83	6.35	50.1	Nervous system disorders
Cogwheel rigidity	5	133.75	15.62	1144.85	Nervous system disorders
Bell's palsy	47	17.48	12.1	25.24	Nervous system disorders
Agraphia	4	106.99	11.96	957.32	Nervous system disorders
Facial nerve disorder	9	17.2	7.44	39.73	Nervous system disorders

Confusional state	458	3.99	3.61	4.4	Psychiatric disorders
Agitation	137	8.18	6.76	9.91	Psychiatric disorders
Disorientation	135	6.39	5.3	7.72	Psychiatric disorders
Mental status changes	152	7	5.86	8.38	Psychiatric disorders
Disorganised speech	3	6.69	1.89	23.7	Psychiatric disorders
Hallucination	38	2.03	1.46	2.83	Psychiatric disorders
Hallucination, auditory	7	4.93	2.2	11.03	Psychiatric disorders
Hallucination, visual	26	9.04	5.79	14.1	Psychiatric disorders
Aggression	14	2.34	1.36	4.04	Psychiatric disorders
Communication disorder	7	5.06	2.26	11.35	Psychiatric disorders
Delirium	86	3.93	3.13	4.93	Psychiatric disorders
Intensive care unit delirium	4	35.66	7.98	159.36	Psychiatric disorders
Flat affect	5	44.58	10.65	186.56	Psychiatric disorders
Bradyphrenia	21	10.22	6.18	16.89	Psychiatric disorders
Bruxism	3	3.82	1.14	12.81	Psychiatric disorders
Impulsive behaviour	3	26.75	5.4	132.53	Psychiatric disorders
Behaviour disorder	4	3.06	1.09	8.6	Psychiatric disorders
Staring	3	6.69	1.89	23.7	Psychiatric disorders
Mutism	3	5.73	1.65	19.95	Psychiatric disorders
Dysphoria	3	4.46	1.31	15.13	Psychiatric disorders
Listless	11	7.95	4.06	15.59	Psychiatric disorders
Renal tubular necrosis	23	5.09	3.26	7.94	Renal and urinary disorders
Incontinence	60	8.24	6.17	11	Renal and urinary disorders
Oliguria	14	3.64	2.08	6.36	Renal and urinary disorders
Urinary incontinence	24	1.82	1.21	2.76	Renal and urinary disorders
Kidney enlargement	3	4.46	1.31	15.13	Renal and urinary disorders
Azotaemia	11	3.82	2.03	7.19	Renal and urinary disorders
Paroxysmal nocturnal haemoglobinuria	3	40.12	6.7	240.13	Renal and urinary disorders
Pleural effusion	139	1.35	1.14	1.6	Respiratory, thoracic and mediastinal disorders
Respiratory failure	167	1.72	1.47	2.01	Respiratory, thoracic and mediastinal disorders
Hypoxia	435	11.35	10.14	12.7	Respiratory, thoracic and mediastinal disorders
Acute respiratory distress syndrome	45	2.09	1.54	2.83	Respiratory, thoracic and mediastinal disorders
Acute respiratory failure	57	2.39	1.83	3.14	Respiratory, thoracic and mediastinal disorders
Tachypnoea	94	8.51	6.75	10.73	Respiratory, thoracic and mediastinal disorders
Apnoea	6	2.92	1.26	6.78	Respiratory, thoracic and mediastinal disorders

Atelectasis	21	3.38	2.15	5.33	Respiratory, thoracic and mediastinal disorders
Respiratory distress	46	1.82	1.35	2.45	Respiratory, thoracic and mediastinal disorders
Pulmonary haemorrhage	21	2.44	1.56	3.82	Respiratory, thoracic and mediastinal disorders
Lung consolidation	7	3.02	1.38	6.6	Respiratory, thoracic and mediastinal disorders
Aspiration	20	3.64	2.28	5.81	Respiratory, thoracic and mediastinal disorders
Pharyngeal haemorrhage	3	3.49	1.05	11.62	Respiratory, thoracic and mediastinal disorders
Pulmonary toxicity	17	1.67	1.02	2.73	Respiratory, thoracic and mediastinal disorders
Respiratory arrest	11	2.25	1.21	4.16	Respiratory, thoracic and mediastinal disorders
Pulmonary alveolar haemorrhage	12	2.63	1.45	4.76	Respiratory, thoracic and mediastinal disorders
Laryngeal oedema	10	3.47	1.8	6.71	Respiratory, thoracic and mediastinal disorders
Lung opacity	8	2.74	1.33	5.68	Respiratory, thoracic and mediastinal disorders
Grunting	3	187.23	9.67	3625.03	Respiratory, thoracic and mediastinal disorders
Respiratory acidosis	3	4.22	1.25	14.27	Respiratory, thoracic and mediastinal disorders
Ecchymosis	15	2.07	1.22	3.5	Skin and subcutaneous tissue disorders
Purpura fulminans	3	13.37	3.34	53.48	Skin and subcutaneous tissue disorders
Eosinophilic cellulitis	3	8.92	2.41	32.94	Skin and subcutaneous tissue disorders
Haemodynamic instability	18	4.12	2.51	6.76	Vascular disorders
Hypotension	870	5.05	4.69	5.43	Vascular disorders
Orthostatic hypotension	27	1.67	1.13	2.46	Vascular disorders
Shock haemorrhagic	14	2.71	1.57	4.7	Vascular disorders
Capillary leak syndrome	18	6.17	3.7	10.31	Vascular disorders
Shock	45	3.47	2.54	4.74	Vascular disorders
Hypoperfusion	4	4.46	1.55	12.85	Vascular disorders
Hypertensive emergency	3	6.69	1.89	23.7	Vascular disorders
Distributive shock	8	7.64	3.48	16.77	Vascular disorders
Ischaemia	5	3.04	1.21	7.67	Vascular disorders

ROR, reporting odds ratio; 95% CI, 95% confidence interval; LL, lower limit; UL, upper limit;

Supplementary Table 7. The Fatality and its Reporting Odds Ratios for High-fatality Adverse Events in CAR-T cell Therapy

Preferred term	No. of deaths	Fatality rate	ROR	95% CI (LL)	95% CI (UL)	System Organ Class	CART-specific AE*
Disseminated intravascular coagulation	46	59.74%	3.50	2.22	5.52	Blood and lymphatic system disorders	Yes
Coagulopathy	38	43.18%	1.79	1.17	2.73	Blood and lymphatic system disorders	Yes
Cytopenia	149	43.82%	1.84	1.49	2.29	Blood and lymphatic system disorders	Yes
Cardio-respiratory arrest	27	81.82%	10.61	4.38	25.70	Cardiac disorders	Yes
Ventricular tachycardia	15	53.57%	2.72	1.29	5.71	Cardiac disorders	Yes
Cardiac arrest	67	83.75%	12.18	6.72	22.07	Cardiac disorders	Yes
Ventricular fibrillation	10	83.33%	11.77	2.58	53.74	Cardiac disorders	Yes
Pulseless electrical activity	18	75%	7.07	2.8	17.81	Cardiac disorders	Yes
Cardiopulmonary failure	7	87.5%	16.48	2.03	133.95	Cardiac disorders	Yes
Pupil fixed	5	100%	25.89	1.43	468.3	Eye disorders	Yes
Mydriasis	5	83.33%	11.77	1.37	100.75	Eye disorders	Yes
Pupillary reflex impaired	4	80%	9.41	1.05	84.24	Eye disorders	Yes
Abdominal compartment syndrome	5	100%	25.89	1.43	468.3	Gastrointestinal disorders	Yes
Intestinal perforation	12	60%	3.53	1.44	8.64	Gastrointestinal disorders	Yes
Hepatic failure	36	70.59%	5.66	3.1	10.34	Hepatobiliary disorders	Yes
Haemophagocytic lymphohistiocytosis	161	61.45%	3.78	2.95	4.85	Immune system disorders	Yes
Systemic candida	25	89.29%	19.64	5.93	65.06	Infections and infestations	Yes
Encephalitis	9	52.94%	2.65	1.02	6.87	Infections and infestations	Yes
Human herpesvirus 6 infection	18	66.67%	4.71	2.12	10.49	Infections and infestations	Yes
Bacteraemia	33	44%	1.85	1.17	2.92	Infections and infestations	Yes
Septic shock	108	74.48%	6.91	4.76	10.05	Infections and infestations	Yes
Staphylococcal infection	44	60.27%	3.58	2.24	5.72	Infections and infestations	Yes
Enterococcal infection	38	71.7%	5.97	3.29	10.86	Infections and infestations	Yes
Candida infection	33	66%	4.58	2.55	8.22	Infections and infestations	Yes
Systemic mycosis	8	66.67%	4.71	1.42	15.64	Infections and infestations	Yes
Fungaemia	15	68.18%	5.05	2.06	12.38	Infections and infestations	Yes
Mucormycosis	15	75%	7.07	2.57	19.44	Infections and infestations	Yes
Klebsiella infection	17	77.27%	8.01	2.95	21.71	Infections and infestations	Yes
Adenovirus infection	15	62.5%	3.92	1.72	8.97	Infections and infestations	Yes
Enterococcal bacteraemia	9	75%	7.06	1.91	26.09	Infections and infestations	Yes
Enterococcal sepsis	6	75%	7.06	1.43	34.99	Infections and infestations	Yes
Stenotrophomonas infection	9	90%	21.19	2.68	167.28	Infections and infestations	Yes
Pseudomonas infection	17	62.96%	4.00	1.83	8.75	Infections and infestations	Yes
Pulmonary mucormycosis	5	100%	25.89	1.43	468.3	Infections and infestations	Yes

Acidosis	16	84.21%	12.56	3.66	43.12	Metabolism and nutrition disorders	Yes
Metabolic acidosis	25	80.65%	9.82	4.03	23.94	Metabolism and nutrition disorders	Yes
Lactic acidosis	31	83.78%	12.18	5.08	29.20	Metabolism and nutrition disorders	Yes
Hypervolaemia	32	50%	2.36	1.44	3.85	Metabolism and nutrition disorders	Yes
Subarachnoid haemorrhage	8	66.67%	4.71	1.42	15.64	Nervous system disorders	Yes
Motor dysfunction	17	50%	2.35	1.20	4.61	Nervous system disorders	Yes
Brain oedema	55	60.44%	3.60	2.37	5.49	Nervous system disorders	Yes
Embolic stroke	5	71.43%	5.88	1.14	30.33	Nervous system disorders	Yes
Clonus	5	71.43%	5.88	1.14	30.33	Nervous system disorders	Yes
Paraparesis	13	52%	2.55	1.16	5.59	Nervous system disorders	Yes
Oliguria	9	64.29%	4.24	1.42	12.65	Renal and urinary disorders	Yes
Respiratory failure	121	72.46%	6.23	4.44	8.76	Respiratory, thoracic and mediastinal disorders	Yes
Hypoxia	154	35.4%	1.29	1.06	1.57	Respiratory, thoracic and mediastinal disorders	Yes
Acute respiratory distress syndrome	38	84.44%	12.81	5.72	28.68	Respiratory, thoracic and mediastinal disorders	Yes
Acute respiratory failure	41	71.93%	6.04	3.39	10.77	Respiratory, thoracic and mediastinal disorders	Yes
Tachypnoea	38	40.43%	1.60	1.06	2.41	Respiratory, thoracic and mediastinal disorders	Yes
Respiratory distress	25	54.35%	2.80	1.57	5.01	Respiratory, thoracic and mediastinal disorders	Yes
Pulmonary haemorrhage	19	90.48%	22.38	5.21	96.11	Respiratory, thoracic and mediastinal disorders	Yes
Aspiration	15	75%	7.07	2.57	19.44	Respiratory, thoracic and mediastinal disorders	Yes
Pulmonary toxicity	14	82.35%	10.99	3.16	38.25	Respiratory, thoracic and mediastinal disorders	Yes
Respiratory arrest	8	72.73%	6.28	1.67	23.67	Respiratory, thoracic and mediastinal disorders	Yes
Pulmonary alveolar haemorrhage	10	83.33%	11.77	2.58	53.74	Respiratory, thoracic and mediastinal disorders	Yes
Haemodynamic instability	12	66.67%	4.71	1.77	12.55	Vascular disorders	Yes
Shock haemorrhagic	11	78.57%	8.63	2.41	30.95	Vascular disorders	Yes
Shock	30	66.67%	4.71	2.54	8.76	Vascular disorders	Yes
Thrombocytopenia	121	39.03%	1.51	1.20	1.90	Blood and lymphatic system disorders	No

Lymphocytosis	7	77.78%	8.24	1.71	39.67	Blood and lymphatic system disorders	No
Splenomegaly	8	80%	9.42	2.00	44.35	Blood and lymphatic system disorders	No
Cardiac failure congestive	6	66.67%	4.71	1.18	18.83	Cardiac disorders	No
Cardiac failure	31	75.61%	7.31	3.58	14.91	Cardiac disorders	No
Myocardial infarction	11	68.75%	5.18	1.80	14.91	Cardiac disorders	No
Acute myocardial infarction	8	61.54%	3.77	1.23	11.52	Cardiac disorders	No
Cardiogenic shock	6	75%	7.06	1.43	34.99	Cardiac disorders	No
Cardiac dysfunction	5	71.43%	5.88	1.14	30.33	Cardiac disorders	No
Ascites	15	62.5%	3.92	1.72	8.97	Gastrointestinal disorders	No
Gastrointestinal haemorrhage	33	61.11%	3.70	2.14	6.40	Gastrointestinal disorders	No
Melaena	7	70%	5.49	1.42	21.24	Gastrointestinal disorders	No
Intestinal ischaemia	7	100%	35.31	2.02	618.35	Gastrointestinal disorders	No
Upper gastrointestinal haemorrhage	5	83.33%	11.77	1.37	100.75	Gastrointestinal disorders	No
Mouth haemorrhage	7	100%	35.31	2.02	618.35	Gastrointestinal disorders	No
Neutropenic colitis	5	71.43%	5.88	1.14	30.33	Gastrointestinal disorders	No
Hepatic steatosis	5	100%	25.89	1.43	468.3	Hepatobiliary disorders	No
Liver disorder	11	50%	2.35	1.02	5.43	Hepatobiliary disorders	No
Hepatotoxicity	17	51.52%	2.5	1.26	4.95	Hepatobiliary disorders	No
Acute graft versus host disease	7	70%	5.49	1.42	21.24	Immune system disorders	No
Sepsis	155	67.39%	4.91	3.72	6.47	Infections and infestations	No
Infection	117	57.64%	3.22	2.44	4.26	Infections and infestations	No
Pneumonia	88	36.97%	1.38	1.06	1.80	Infections and infestations	No
Staphylococcal sepsis	5	71.43%	5.88	1.14	30.33	Infections and infestations	No
Bacterial infection	20	51.28%	2.48	1.32	4.65	Infections and infestations	No
Fungal infection	34	72.34%	6.17	3.25	11.69	Infections and infestations	No
Cytomegalovirus infection	24	53.33%	2.69	1.50	4.84	Infections and infestations	No
Neutropenic sepsis	11	57.89%	3.24	1.30	8.05	Infections and infestations	No
Aspergillus infection	28	73.68%	6.6	3.20	13.59	Infections and infestations	No
Pneumonia fungal	13	54.17%	2.78	1.25	6.21	Infections and infestations	No
Pneumonia pseudomonal	7	70%	5.49	1.42	21.24	Infections and infestations	No
Gastrointestinal infection	12	70.59%	5.65	1.99	16.04	Infections and infestations	No
Bacterial sepsis	10	83.33%	11.77	2.58	53.74	Infections and infestations	No
Pseudomonal sepsis	7	63.64%	4.12	1.21	14.07	Infections and infestations	No
Progressive multifocal leukoencephalopathy	15	55.56%	2.94	1.38	6.29	Infections and infestations	No
Fungal sepsis	5	100%	25.89	1.43	468.3	Infections and infestations	No
Covid-19	63	40.13%	1.58	1.15	2.17	Infections and infestations	No
Pneumonia aspiration	18	75%	7.07	2.8	17.81	Infections and infestations	No
Covid-19 pneumonia	22	62.86%	3.99	2.01	7.92	Infections and infestations	No
Encephalitis cytomegalovirus	4	100%	21.18	1.14	393.49	Infections and infestations	No

Tumour lysis syndrome	31	44.29%	1.87	1.17	3.00	Metabolism and nutrition disorders	No
Electrolyte imbalance	9	52.94%	2.65	1.02	6.87	Metabolism and nutrition disorders	No
Hyperkalaemia	14	60.87%	3.66	1.59	8.46	Metabolism and nutrition disorders	No
Hypercalcaemia	9	56.25%	3.03	1.13	8.13	Metabolism and nutrition disorders	No
Failure to thrive	9	75%	7.06	1.91	26.09	Metabolism and nutrition disorders	No
Hyperammonaemia	4	80%	9.41	1.05	84.24	Metabolism and nutrition disorders	No
Haemorrhage intracranial	13	72.22%	6.12	2.18	17.18	Nervous system disorders	No
Posterior reversible encephalopathy syndrome	6	66.67%	4.71	1.18	18.83	Nervous system disorders	No
Cerebral haemorrhage	26	86.67%	15.32	5.35	43.91	Nervous system disorders	No
Cerebral infarction	9	56.25%	3.03	1.13	8.13	Nervous system disorders	No
Leukoencephalopathy	10	58.82%	3.36	1.28	8.84	Nervous system disorders	No
Brain injury	5	83.33%	11.77	1.37	100.75	Nervous system disorders	No
Cerebral ischaemia	4	80%	9.41	1.05	84.24	Nervous system disorders	No
Acute kidney injury	92	41.63%	1.68	1.29	2.2	Renal and urinary disorders	No
Renal failure	57	77.03%	7.92	4.60	13.61	Renal and urinary disorders	No
Renal impairment	16	50%	2.35	1.18	4.71	Renal and urinary disorders	No
Nephropathy toxic	11	64.71%	4.32	1.60	11.67	Renal and urinary disorders	No
Anuria	8	88.89%	18.83	2.36	150.6	Renal and urinary disorders	No
Pulmonary oedema	23	48.94%	2.26	1.27	4.00	Respiratory, thoracic and mediastinal disorders	No
Haemoptysis	7	63.64%	4.12	1.21	14.07	Respiratory, thoracic and mediastinal disorders	No
Circulatory collapse	8	72.73%	6.28	1.67	23.67	Vascular disorders	No
Haemorrhage	18	66.67%	4.71	2.12	10.49	Vascular disorders	No
Venoocclusive disease	6	85.71%	14.12	1.70	117.33	Vascular disorders	No

ROR, reporting odds ratio; 95% CI, 95% confidence interval; LL, lower limit; UL, upper limit; AE, adverse event.

*indicates whether it was identified as a CAR-T therapy related adverse event in the disproportionality analysis

Supplementary Table 8. The Fatality and its Reporting Odds Ratios for High-fatality CAR-T Therapy related Adverse Events at the System-organ Level.

System Organ Class	No. of deaths	Fatality rate	ROR	95% CI (LL)	95% CI (UL)
Respiratory, thoracic and mediastinal disorders	483	52.22%	2.63	2.31	2.99
Infections and infestations	424	67.3%	4.96	4.2	5.87
Blood and lymphatic system disorders	233	46.14%	2.03	1.7	2.42
Immune system disorders	161	61.45%	3.78	2.95	4.85
Cardiac disorders	144	77.84%	8.34	5.89	11.8
Metabolism and nutrition disorders	104	68.87%	5.24	3.71	7.4
Nervous system disorders	103	58.52%	3.34	2.47	4.51
Vascular disorders	53	68.83%	5.21	3.22	8.45
Hepatobiliary disorders	36	70.59%	5.66	3.1	10.34
Gastrointestinal disorders	17	68%	5	2.16	11.6
Eye disorders	14	87.5%	16.49	3.75	72.55
Renal and urinary disorders	9	64.29%	4.24	1.42	12.65

ROR, reporting odds ratio; 95% CI, 95% confidence interval; LL, lower limit; UL, upper limit;

Supplementary Table 9. The Fatality and its Reporting Odds Ratios for Low-fatality Adverse Events in CAR-T cell Therapy

Preferred term	No. of deaths	Fatality rate	ROR	95% CI (LL)	95% CI (UL)	System Organ Class	CART-specific AE
B-cell aplasia	15	15.62%	0.44	0.25	0.76	Blood and lymphatic system disorders	Yes
Tachycardia	82	22.53%	0.68	0.53	0.87	Cardiac disorders	Yes
Cardiorenal syndrome	3	6.52%	0.16	0.05	0.53	Cardiac disorders	Yes
Cytokine release syndrome	1123	19.35%	0.53	0.49	0.56	Immune system disorders	Yes
Cytokine storm	6	12%	0.32	0.14	0.75	Immune system disorders	Yes
Immunodeficiency	5	11.63%	0.31	0.12	0.79	Immune system disorders	Yes
Hypoalbuminaemia	10	6.76%	0.17	0.09	0.32	Metabolism and nutrition disorders	Yes
Hypokalaemia	7	6.54%	0.16	0.08	0.35	Metabolism and nutrition disorders	Yes
Hyponatraemia	8	9.41%	0.24	0.12	0.51	Metabolism and nutrition disorders	Yes
Hypocalcaemia	3	5.17%	0.13	0.04	0.41	Metabolism and nutrition disorders	Yes
Hypomagnesaemia	0	0%	0.02	0	0.37	Metabolism and nutrition disorders	Yes
Hypophosphataemia	4	6.06%	0.15	0.06	0.42	Metabolism and nutrition disorders	Yes
Aphasia	46	13.69%	0.37	0.27	0.51	Nervous system disorders	Yes
Tremor	45	12.47%	0.33	0.24	0.46	Nervous system disorders	Yes
Neurotoxicity	387	20.29%	0.59	0.52	0.66	Nervous system disorders	Yes
Somnolence	49	18.92%	0.55	0.4	0.75	Nervous system disorders	Yes
Headache	34	8.29%	0.21	0.15	0.3	Nervous system disorders	Yes
Seizure	38	20.99%	0.62	0.44	0.89	Nervous system disorders	Yes
Dysgraphia	13	13.98%	0.38	0.21	0.69	Nervous system disorders	Yes
Memory impairment	15	10.71%	0.28	0.16	0.48	Nervous system disorders	Yes
Facial paralysis	7	10.45%	0.27	0.13	0.6	Nervous system disorders	Yes
Speech disorder	7	15.22%	0.42	0.19	0.94	Nervous system disorders	Yes
ICANS	517	23.26%	0.7	0.64	0.78	Nervous system disorders	Yes
Parkinsonism	9	15.79%	0.44	0.22	0.9	Nervous system disorders	Yes
Bell’s palsy	4	8.51%	0.22	0.08	0.61	Nervous system disorders	Yes
Confusional state	85	18.56%	0.53	0.42	0.68	Psychiatric disorders	Yes
Disorientation	25	18.52%	0.53	0.35	0.82	Psychiatric disorders	Yes
Bradyphrenia	1	4.76%	0.12	0.02	0.88	Psychiatric disorders	Yes
Incontinence	9	15%	0.41	0.2	0.84	Renal and urinary disorders	Yes
Hypotension	226	25.98%	0.82	0.71	0.96	Vascular disorders	Yes
Orthostatic hypotension	1	3.7%	0.09	0.01	0.67	Vascular disorders	Yes
Neutropenia	115	25.44%	0.8	0.65	0.99	Blood and lymphatic system disorders	No
Anaemia	37	15.1%	0.42	0.29	0.59	Blood and lymphatic system disorders	No
Nausea	26	10.12%	0.26	0.18	0.4	Gastrointestinal disorders	No
Vomiting	22	13.75%	0.37	0.24	0.59	Gastrointestinal disorders	No
Diarrhoea	30	12.66%	0.34	0.23	0.5	Gastrointestinal disorders	No
Influenza	9	16.98%	0.48	0.23	0.99	Infections and infestations	No
Decreased appetite	16	7.48%	0.19	0.11	0.32	Metabolism and nutrition disorders	No
Muscular weakness	10	16.39%	0.46	0.23	0.91	Musculoskeletal and connective tissue disorders	No

Myalgia	5	4.81%	0.12	0.05	0.29	Musculoskeletal and connective tissue disorders	No
Arthralgia	7	8.97%	0.23	0.11	0.5	Musculoskeletal and connective tissue disorders	No
Dizziness	5	8.47%	0.22	0.09	0.54	Nervous system disorders	No
Balance disorder	5	13.51%	0.37	0.14	0.94	Nervous system disorders	No
Neuropathy peripheral	8	15.38%	0.43	0.2	0.91	Nervous system disorders	No
Paraesthesia	2	7.69%	0.2	0.05	0.83	Nervous system disorders	No
Insomnia	2	5.88%	0.15	0.04	0.61	Psychiatric disorders	No
Cough	14	16.28%	0.46	0.26	0.81	Respiratory, thoracic and mediastinal disorders	No
Rash	6	8.96%	0.23	0.1	0.53	Skin and subcutaneous tissue disorders	No
Erythema	2	7.41%	0.19	0.04	0.79	Skin and subcutaneous tissue disorders	No
Skin lesion	3	9.09%	0.24	0.07	0.77	Skin and subcutaneous tissue disorders	No
Deep vein thrombosis	4	11.43%	0.3	0.11	0.86	Vascular disorders	No

ROR, reporting odds ratio; 95% CI, 95% confidence interval; LL, lower limit; UL, upper limit; ICANS, Immune effector cell-associated neurotoxicity syndrome; AE, adverse event.

*indicates whether it was identified as a CAR-T therapy related adverse event in the disproportionality analysis

Supplementary Table 10. The Fatality and its Reporting Odds Ratios for Low-fatality CAR-T Therapy related Adverse Events at the System-organ Level.

System Organ Class	No. of deaths	Fatality rate	ROR	95% CI (LL)	95% CI (UL)
Nervous system disorders	1171	19.11%	0.52	0.48	0.55
Immune system disorders	1134	19.23%	0.52	0.49	0.56
Vascular disorders	227	25.31%	0.79	0.68	0.92
Psychiatric disorders	111	18.08%	0.52	0.42	0.63
Cardiac disorders	85	20.73%	0.61	0.48	0.78
Metabolism and nutrition disorders	32	6.21%	0.15	0.11	0.22
Blood and lymphatic system disorders	15	15.62%	0.44	0.25	0.76
Renal and urinary disorders	9	15%	0.41	0.2	0.84

ROR, reporting odds ratio; 95% CI, 95% confidence interval; LL, lower limit; UL, upper limit;

Supplementary Table 11. Reporting proportions of CRS and ICANS according to CAR-T drug type.

Drug type	Total cases	CRS† count (proportion)	ICNAS# count (proportion)
Axicabtagene-ciloleucel	5919	3106 (52.48%)	2485 (41.98%)
Tisagenlecleucel	3055	1302 (42.62%)	661 (21.64%)
Lisocabtagene-maraleucel	377	166 (44.03%)	145 (38.46%)
Brexucabtagene autoleucel	1138	601 (52.81%)	536 (47.10%)
Idecabtagene vicleucel	659	434 (65.86%)	167 (25.34%)
Ciltacabtagene-autoleucel	1363	244 (17.90%)	136 (9.98%)

CRS, cytokine release syndrome; ICANS, Immune effector cell-associated neurotoxicity syndrome

† Including the preferred terms cytokine release syndrome and cytokine storm.

# Including the preferred terms immune effector cell-associated neurotoxicity syndrome and neurotoxicity.
